# Resisting aggression in social contexts: The influence of life-course persistent antisocial behavior on behavioral and neural responses to social feedback

**DOI:** 10.1016/j.nicl.2022.102973

**Published:** 2022-02-26

**Authors:** Ilse H. van de Groep, Marieke G.N. Bos, Lucres M.C. Jansen, Desana Kocevska, Anika Bexkens, Moran Cohn, Lieke van Domburgh, Arne Popma, Eveline A. Crone

**Affiliations:** a Erasmus School of Social and Behavioral Sciences, Erasmus University Rotterdam, The Netherlands; b Leiden Institute for Brain and Cognition, Leiden University, The Netherlands; c Department of Developmental and Educational Psychology, Institute of Psychology, Leiden University, The Netherlands; d Department of Child and Adolescent Psychiatry & Psychosocial Care, Amsterdam University Medical Center, Amsterdam, The Netherlands; e Department of Sleep and Cognition, Netherlands Institute for Neuroscience, An Institute of the Royal Netherlands Society for Arts and Sciences, Amsterdam, The Netherlands; f GGZ Delfland, Center for Psychiatry, Department of Child and Adolescent Psychiatry, Delft, The Netherlands; g Quality of Care & Innovation, Child and Adolescent Psychiatry & Psychosocial Care, Pluryn Nijmegen, The Netherlands

**Keywords:** Antisocial behavior, Aggression, Social evaluation, fMRI, Antisocial developmental trajectories

## Abstract

Early adulthood has long been recognized as a potential turning point for the development of antisocial behavior, due to changes in social contexts and ongoing psychological and neurobiological maturation. However, it remains unclear how different developmental trajectories of antisocial behavior, their neural underpinnings, and individual differences in psychopathic traits may help explain the distinct developmental outcomes of individuals who persist in or desist from antisocial behavior in early adulthood - such as how they respond to others in social contexts. Therefore, in the current study, young adults (aged 18–30, 68% male) with a persistent or desistant antisocial trajectory (N = 54), as well as healthy controls (N = 39), completed the Social Network Aggression Task, during which they received positive, neutral, or negative feedback on a personal profile and got the opportunity to retaliate by blasting a loud noise. On a behavioral level, results indicated that in all groups, negative peer feedback evoked higher retaliatory aggression, compared to positive and neutral feedback. On a neural level, when receiving social feedback, individuals with persistent or desistent trajectories showed both similar and dissociable patterns of neural activity; desisting and persisting trajectory groups showed higher activity in the Insula, and the desisting trajectory group showed higher activity in dlPFC. Finally, when participants retaliated, they showed increased dlPFC and ACC activity following positive relative to neutral and negative feedback, where ACC activity correlated most strongly with inhibition of retaliatory responses in the desisting trajectory group. Together, these findings provide novel insights in dissociable patterns of brain activity that may increase our understanding of the mechanisms underlying different developmental trajectories of antisocial behavior.

## Highlights

• Negative social feedback (vs positive / neutral) evoked more retaliatory aggression.
• Persistent and desistent antisocial development associated with similar and dissociable neural activity:
• During social feedback processing: Increased Insula (both groups) or dlPFC activation (desisters)
• During retaliation: Increased dlPFC and ACC activity after positive feedback.
• During retaliation: ACC activity correlated with inhibition of retaliation (desisters)

## 1 Introduction

Antisocial behavior is defined as behavior that violates the rights or wellbeing of others, and often conflicts with age-appropriate norms and rules (Frick et al., 2018). Children who show antisocial behavior at an early age are at risk of developing persistent antisocial behavior (i.e., antisocial behavior that continues throughout adolescence and adulthood; also known as life-course or early-onset persistent antisocial behavior Moffitt, 1993, 2018), and for poorer outcomes in various life domains related to health, finances and social relationships (Moffitt, 2018, Pulkkinen et al., 2009). However, only a small group of children with an early onset of antisocial behavior actually show persistent antisocial behavior throughout their lives, and in general, desistance from antisocial development is the norm (Bersani and Doherty, 2018, Moffitt, 2018). In line with this idea, longitudinal research has identified people who show desistent, *childhood-limited* antisocial behavior, who are likewise characterized by high levels of conduct problems early in life (Aguilar et al., 2000, Bevilacqua et al., 2018, Fairchild et al., 2013, Odgers et al., 2007, Odgers et al., 2008). These children typically desist from antisocial behavior in adolescence and early adulthood, and show better life outcomes in the majority of (but not all) domains compared to the life-course persistent and adolescence limited groups (Bevilacqua et al., 2018, Carlisi et al., 2020, Carlisi et al., 2021, Odgers et al., 2007, Odgers et al., 2008).

Hence, while early developmental experiences are considered important to the etiology and maintenance of antisocial behavior (Moffitt, 2018), an early age of onset of antisocial behavior itself is not a strong predictor of antisocial development, since it has been associated with both relatively adverse outcomes (in the case of life-course persistent antisocial behavior), and relatively positive outcomes (in the case of childhood-limited antisocial behavior, see Bevilacqua et al., 2018; but see Carlisi et al., 2021, Moffitt et al., 2002). Therefore, it is important to identify possible candidate mechanisms that allow us to gain a better understanding why such developmental outcome differences (i.e., desisting or persisting antisocial trajectories) arise between groups with an early onset. Here, we propose that investigating different behavioral and neural responses to social rejection between these subgroups may contribute to a better understanding of the mechanisms that underlie persistence and desistance of antisocial behaviors in early adulthood.

### 1.1 Behavioral and neural responses to social rejection in adolescence and early adulthood

An emerging body of evidence suggests that social context-related factors, such as social (peer) rejection, may be important to understand why differences in developmental outcomes emerge between persisters and desisters in early adulthood (Moffitt, 1993; Monahan et al., 2009, Veenstra et al., 2009). Changes in social context can either provide positive developmental opportunities and desistance of antisocial behavior, or aggravate existing patterns of antisocial behavior (Cyr et al., 2020, Hyde et al., 2018). In the transition from childhood to adolescence, there is a reorientation in the social context toward peers (Crone and Dahl, 2012) and adolescents become increasingly susceptible to peer influence (Prinstein and Dodge, 2008). Accordingly, peers often have a profound influence on the development of antisocial behavior (Monahan et al., 2009; Moffitt, 1993), which manifests itself through peer selection (i.e., who people tend to affiliate with; e.g. antisocial or prosocial peers; Kandel, 1978, Monahan et al., 2009) and peer socialization (i.e., how affiliation and social interactions influence subsequent (anti-)social behavior; Monahan et al., 2009, Prinstein and Dodge, 2008)). The transition from adolescence to early adulthood is also characterized by a shift in social context, marked by taking on new social roles and establishing long-term relationships (Arnett, 2007), more freedom and less social control (Arnett, 2005). Generally, when people develop into young adults, they become less susceptible to effects of peer selection and socialization (Monahan et al., 2009, Arnett, 2007). However, there are also remarkable individual differences in the speed of, and the actual development of this capacity (Monahan et al., 2009).

Possibly, such individual differences may coincide with persistent or desistent developmental trajectories. In children and adolescents who display life-course persistent antisocial behavior, repeated social rejection often triggers maladaptive behaviors such as aggression (Veenstra et al., 2009), likely in an attempt to gain social acceptance or maintain positive self-views (David and Kistner, 2000, van de Groep et al., 2021, Veenstra et al., 2009). This aggressive behavior, in turn, is likely to elicit more social rejection by prosocial peers, and affiliation with deviant peers, which may result in a vicious cycle of maladaptive antisocial behavior throughout development (Veenstra et al., 2009) In contrast, people who desist from antisocial behavior may be more likely to have positive, prosocial experiences throughout development that allow them to deflect from antisocial responses (Cyr et al., 2020, Hyde et al., 2018). However, much less is known about whether these group differences extend into early adulthood (Hyde et al., 2018, Moffitt et al., 2002).

To study (immediate) behavioral and neural responses to social rejection, previous studies in adults have used social exclusion (Cyberball; e.g. Chester et al., 2014) and social feedback paradigms (e.g. Social Network Aggression Task (SNAT); Achterberg et al., 2016). In the SNAT, participants are evaluated on their personal profile and receive acceptance, neutral or rejection feedback by age-matched peers. Subsequently, they can blast a noise towards the peer in response to the feedback (see also Chester et al., 2014 for a similar approach). Rejection feedback was associated with longer noise blasts (Achterberg et al., 2016, Chester et al., 2014, van de Groep et al., 2021), which is indicative of more retaliatory / aggressive responses.

Interestingly, the SNAT has also been used to examine the neural underpinnings of feedback processing and retaliatory responses in children, adolescents and young adults. On a neural level, social rejection and acceptance feedback led to increased activity in the Insula, ACC and Medial Prefrontal Cortex (mPFC), regions often associated with saliency processing (Achterberg et al., 2016, Achterberg et al., 2017, Achterberg et al., 2018, Achterberg et al., 2020). Increased neural activation in the dlPFC after negative feedback (relative to positive or neutral) has been associated with less aggressive behavior after negative feedback (Achterberg et al., 2016, Achterberg et al., 2017, Achterberg et al., 2018, Achterberg et al., 2020, van de Groep et al., 2021). These findings are consistent with other social feedback paradigms that demonstrated a causal relation between dlPFC stimulation through transcranial magnetic stimulation and aggression following rejection (Riva et al., 2015).

It has recently been suggested that the aforementioned brain areas (i.e., Insula, ACC, mPFC and dlPFC) may be important for differentiating between positive and negative development opportunities in early adulthood (Taber-Thomas and Pérez-Edgar, 2015), and they have been implicated in the stability and severity of antisocial behavior (Alegria et al., 2016, Aoki et al., 2014, Carlisi et al., 2020, Carlisi et al., 2021, Dugré et al., 2020, Fairchild et al., 2011, Yang and Raine, 2009). More specifically, social changes in early adulthood are accompanied by continuous changes in brain function and structure (Herting et al., 2018, Tamnes et al., 2017), and the interaction between the emerging social context and neural development may give rise to developmental opportunities and vulnerabilities (Taber-Thomas and Pérez-Edgar, 2015). Moreover, recent neuroimaging studies indicate that life-course persistent antisocial behavior is characterized by abnormal functional and structural development of both cortical and subcortical brain areas, whereas adolescence-limited and childhood-limited antisocial behavior are not (Fairchild et al., 2011, Carlisi et al., 2020, Carlisi et al., 2021). Together, these findings suggest that investigating functional imaging during social rejection may further elucidate possible mechanisms underlying different developmental trajectories of antisocial behavior into early adulthood.

### 1.2 Accounting for heterogeneity in antisocial behavior: individual differences in psychopathic traits

Over the past few years, it has become increasingly clear that aggression is heterogeneous, in its causes, underlying motivations and expression (Girard et al., 2019). Accordingly, researchers have argued against a categorical approach of investigating antisocial and aggressive behavior in individuals who desist or persist in these behavioral profiles, and have argued for a more dimensional perspective on psychopathology, which allows for more nuanced approaches to investigate individual differences (Garvey et al., 2016, Insel et al., 2010).

One factor that has repeatedly been linked to differences in frequency, severity and persistence of aggressive behavior in social contexts is psychopathy (Blair, 2015, Brennan et al., 2018). For instance, higher levels of psychopathic traits have been associated with aberrant processing during social rejection, and psychopathic trait levels moderated the links between social rejection processing and subsequent self-reported emotional and behavioral responses, such as anger and aggression (Brennan et al., 2018). However, several interrelated dimensions of psychopathy may differentially influence aggressive behavior and its underlying behavioral and neural underpinnings. Indeed, Grandiose-Manipulative interpersonal characteristics (marked by lying, manipulating and a grandiose sense of self-worth), Impulsive-Irresponsible traits (characterized by impulsivity and irresponsibility) and Callous-Unemotional traits (characterized by a lack of empathy, remorse and shallow affect; Andershed et al., 2002) have all been differently associated with aggression (Jambroes et al., 2018, Orue et al., 2016, Orue and Andershed, 2015) and with altered brain structure and function in the ACC, Insula and dlPFC (Poeppl et al., 2019, Yang and Raine, 2009). Hence, considering individual differences in psychopathic traits may further elucidate why young adults behave aggressively in social contexts.

### 1.3 The current study

In this pre-registered study, we examined 94 young adults (aged 18–30) who were subtyped according to their history of antisocial behavior as showing (1) persistent antisocial behavior, (2) desistent antisocial behavior or (3) no history of antisocial behavior (henceforth referred to as the control group), with two aims.

Our first aim was to examine (the neural correlates of) aggression regulation following social rejection in early adulthood comparing individuals with different types of antisocial profiles. On a behavioral level, we hypothesized that (1a) across all participants, social rejection results in stronger aggressive responses than positive or neutral social feedback (Achterberg et al., 2016, van de Groep et al., 2021). When comparing groups, we expected that (1b) social rejection results in increased aggression in persisters when compared to desisters and controls (Chester et al., 2014, Achterberg et al., 2016). Second, on a neural level, across all participants, we (2a) expected increased brain activation in the Insula and Anterior Cingulate Cortex (ACC) following positive and negative feedback, when compared to neutral feedback (Achterberg et al., 2016). When comparing groups (2b), we expected that these effects would be stronger in persisters when compared to desisters and controls (Achterberg et al., 2016). Third, we hypothesized that (3a) across all participants, less aggression would be related to increased dlPFC activity, especially during negative feedback (Achterberg et al., 2016). When comparing groups, we expected that (3b) dlPFC activity would be stronger in desisters and controls when compared with persisters (Achterberg et al., 2016). Finally, when considering brain-behavior associations we expected that (3c) the aforementioned relationship between dlPFC activity and aggression would be more negative in desisters and controls when compared to persisters.

The second aim of this study was to examine whether behavioral and neural responses to social rejection differ depending on levels of psychopathic traits. On a behavioral level, we hypothesized that (1c) the three psychopathic trait dimensions (Grandiose-Manipulative, Callous-Unemotional, Impulsive-Irresponsible) are differentially related to aggressive responses following negative feedback. In addition, on a neural level, we expected that (2c) activity in the Insula and ACC are differentially related to the three psychopathic traits (see supplement for the specific behavioral and neural hypotheses for each dimension). The hypotheses, the design and analysis plan were pre-registered prior to data analysis and are available on the Open Science Framework (https://osf.io/d6fku/).

## 2 Methods

### 2.1 Participants

The current study was part of a larger longitudinal study on the development of antisocial behavior from late childhood to early adulthood in the Netherlands (Cohn et al., 2012, Cohn et al., 2013 Cohn et al., 2015a, Cohn et al., 2015b Cohn et al., 2016a; Cohn et al., 2016b; Pape et al., 2015; van Domburgh, 2009a, van Domburgh et al., 2009b, van Domburgh et al., 2011, van Domburgh et al., 2019 Tielbeek, 2018), called ‘RESIST’ (see Figure S1A for an overview of the five different time points, T1 (2003–2006, mean age 10.9 (SD = 1.4)), T2 (2004–2008, mean age 11.4 (SD = 1.5)), T3 (2005–2008, mean age 13.1 (SD = 1.5)), T4 (2010–2012, mean age 17.6 (SD = 1.4)), T5 (2019–2021, mean age 25.5 (SD = 1.7))). For the current study, we approached participants from the original sample (*N* = 364, prioritizing participants who had participated in and up to the previous wave (T4: N = 130), resulting in a sample of 74 participants (see Figure S1A-B). Of these 74 individuals, 55 completed the MRI protocol. Demographic and clinical data did not differ between participants included and excluded for the MRI session (see supplemental methods and Table S1), except for IQ scores, which were higher in the included than the excluded group. In addition, we recruited 40 healthy controls, without a history of antisocial behavior, who also completed the same measures and MRI protocol (see van de Groep et al., 2021). Note that the current study primarily reports cross-sectional data, but uses longitudinal data to determine whether participants desisted or persisted in antisocial behavior (see section 2.2.3).

All participants were screened for fMRI contra-indications and had normal to corrected vision. Participants were excluded from fMRI analyses in case they did not perform or complete the task (*N_control_* = 1, *N_cases_ = 0*), if the MRI data was corrupted (*N_control_* = 1, *N_cases_ = 0*) or showed excessive head motion (>3mm; *N_control_* = 3; *N_cases_* = 1), resulting in final fMRI sample of 35 controls and 53 cases (42 desisters and 11 persisters), respectively. Head motion did not differ between individuals from the control (*M* = 0.095, *SD* = 0.052), desister (*M* = 0.087, *SD* = 0.060) or persister groups (*M* = 0.105, *SD* = 0.102), *F*(2, 93) = 0.43, *p* = .67. Analyses on behavioral results were conducted for those participants who completed the task and required questionnaires (*N_control_* = 39 and *N_cases_* = 54 (42 desisters and 12 persisters), respectively). See Table 1 for an overview of the descriptive data (total sample and sub-groups).

**Table 1:** Sample description, group comparisons and reliability estimates.

	Group			
Measure	Desister (n = 42)	Persister (n = 12)	Control (n = 39)	Statistical comparison
Gender [*n* males/females]	36/6	11/1	16/23	
Age [*M* (*SD*)]	26.20 (1.63)	26.62 (1.13)	22.7 (3.07)	*F* (2,90) = 26.42, *p* < .001^a^
Education [*n*]				*Χ*^2^ = 9.38, *p* = 0.15
Vocational	25	6	16	
College	9	2	18	
University	4	1	4	
Other	4	3	1	
IQ [*M* (*SD*)]^1^	103.47 (14.13)	100.87 (11.08)	107.47 (11.64)	*F* (2,90) = 1.65, *p* = .19
YPI Total psychopathic traits [*M* (*SD*)]^2^	84.91 (16.37)	106.67 (19.51)	87.57 (9.72)	*F* (2,90) = 10.85, *p* < .001^b^
YPI Callous-Unemotional Traits [*M* (*SD*)]	28.77 (3.64)	33.5 (5.03)	29.18 (3.09)	*F* (2,90) = 8.21, *p* < .001^b^
YPI Grandiose-Manipulative Traits [*M* (*SD*)]	27.53 (7.47)	37.33 (11.16)	28.29 (4.99)	*F* (2,90) = 9.20, *p* < .001^b^
YPI Impulsive-Irresponsible Traits [*M* (*SD*)]	28.59 (7.53)	35.83 (7.19)	30.03 (5.70)	*F* (2,90) = 5.34, *p* = .006^b^
Cronbach’s Alpha	Cases		Controls	
YPI Total	0.92		0.81	
YPI Callous-Unemotional Traits	0.52		0.39	
YPI Grandiose-Manipulative Traits	0.91		0.78	
YPI Impulsive-Irresponsible Traits	0.86		0.77	

*Note.* IQ, estimated IQ based on two subscales of the Wechsler Adult Intelligence Scale-IV (Similarities and Block Design), YPI = Youth Psychopathic traits Inventory.

a Significant differences between controls and desisters, and controls and persisters

b Significant differences between persisters and desisters, and persisters and controls

1 Note that for three participants who completed the fMRI session (n*_perister_* = 1, n*_desister_* = 2), the IQ tests at T5 were not completed. Therefore, we estimated these scores using multiple imputation based on the other variables reported in this table, as well as prior IQ scores (T4).

2 Note that for two participants who completed the fMRI session (n*_perister_* = 1, n*_desister_* = 1), the YPI was not completed. Therefore, we estimated these scores using multiple imputation, based on the other variables reported in this table, as well as prior IQ scores (T4).

The study protocol was approved by the VU University Medical Center Medical Ethical Committee (registration number 2009.268 - NL28844.029.09), with local approval from the Leiden Institute for Brain and Cognition. All subjects gave written informed consent in accordance with the Declaration of Helsinki. After completing the experiment, participants were debriefed about the aim of the study and received a financial reimbursement for their participation (75 euros for controls, 100 euros for cases).

### 2.2 Materials

#### 2.2.1 Social Network Aggression Task

**Fig. 1:**
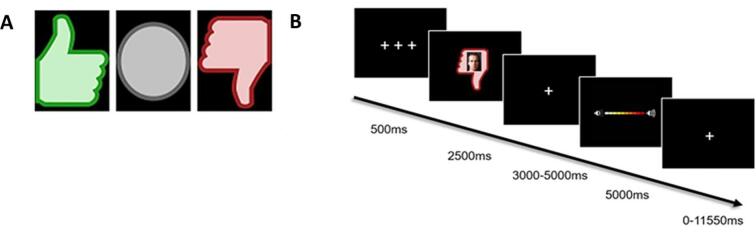
(A) Participants received Positive, Neutral and Negative feedback from same-aged peers. (B) Schematic representation of a Negative feedback trial in the Social Network Aggression Task (SNAT).

To investigate the neural basis of social evaluation and subsequent aggressive responses, we used the Social Network Aggression Task (Achterberg et al., 2016). During this task, participants received social feedback (Positive, Negative, Neutral) from unknown same-aged peers, based on a personal profile completed by the participants prior to the experiment. Social feedback valence was signaled by different icons (green thumbs up for Positive feedback, grey circle for Neutral feedback, red thumbs down for Negative feedback; see Fig. 1A), with superimposed neutral pictures of same-aged peers. After receiving social feedback, participants were asked to respond to the evaluations by sending hypothetical noise blasts to the same-aged peers. Participants were instructed to press the button always, but could control the loudness of the noise blast with a button press. A longer button press corresponded with a longer noise blast duration (i.e., louder white noise). Noise blast duration was visualized by a volume bar (see Fig. 1B for a schematic trial representation). The SNAT consisted of three blocks of 20 trials (60 in total, van de Groep et al., 2021), with three social feedback conditions (i.e., Neutral, Positive, Negative) being semi-randomized across these blocks. Participants could not receive feedback from the same type more than three times in a row. Trial order and jitter timing were optimized using Optseq2 (Dale, 1999).

#### 2.2.2 Youth Psychopathic Traits Inventory (YPI).

Psychopathic traits were assessed using the Youth Psychopathy Inventory (Andershed et al., 2002), a 50-item self-report questionnaire that distinguishes three trait dimensions: Grandiose-Manipulative, Callous-Unemotional, and Impulsive-Irresponsible traits. Although the questionnaire was originally developed to assess psychopathic traits in adolescents, the YPI has also been validated in young adults (see e.g. Campbell et al., 2009, Neumann and Pardini, 2012). Each item is scored on a 4-point Likert scale (1 = does not apply at all, to 4 = applies very well). For both samples, the reliability of the total YPI score, Grandiose-Manipulative and Impulsive-Irresponsible subscales was good to excellent, and reliability of Callous-Unemotional traits was poor for both samples. Total and dimensional sub scores are displayed in Table 1.

#### 2.2.3 Antisocial behavior

All participants with a history of antisocial behavior were arrested by the police before the age of 12. Hence, in the current sample, all individuals with a history of antisocial behavior showed an early onset of such behavior (in the form of a convicted criminal offense, but not necessarily in the form of a disruptive behavior disorder (DBD) (see Table S5), and none of them could be characterized as showing adolescence-limited antisocial behavior. Participants with a history of antisocial behavior were subtyped into different developmental trajectories using diagnostic interviews conducted at ages 14-20 (T4) and ages 21-29 (T5). DBD diagnoses were determined using the National Institute of Mental Health DISC-IV (Shaffer et al., 2000). Antisocial personality disorder was determined by using the MINI-PLUS (Lecrubier et al., 1997), a brief structured diagnostic interview to diagnose psychiatric disorders according to the DSM-IV. Participants were classified as showing persistent antisocial behavior when they received a diagnosis of disruptive behavior disorder at wave 4 (T4) of the longitudinal study, and / or antisocial personality disorder at wave 5 (T5). Of the 54 participants who completed the experimental task, 12 were classified as persister, and 42 as desister (see Figure S1B). One participant did not complete the MINI and could not be classified. Hence, this participant was excluded from all analyses involving subgroup comparisons.

### 2.3 Procedure

Prior to participation, participants received information about the study by telephone and through a digital information letter. Participants in the control sample completed the whole procedure in one session (June – September 2019, van de Groep et al., 2021). For participants in the ‘case’ sample, data collection (for the fifth timepoint, T5) was split across two sessions (a ‘home visit’ and scan session). Both aforementioned questionnaires (i.e., YPI and MINI) were administered during the home visit. Given that data collection for this sample was ongoing during the outbreak of the COVID-19 pandemic, these ‘home visits’ were conducted at participants’ homes between November 2019 and March 13th 2020; and subsequently conducted through skype for business between March 14th 2020 and February 2021. The only other procedural difference was that for the part of the sample who participated after March 13th 2020, IQ tests were completed during the MRI session, instead of the ‘home visit’ session.

After signing informed consent, all participants filled out several questionnaires prior to the scanning session. During the scanning session, participants first received instructions about the tasks and performed practice versions of the fMRI tasks. Since the current study was part of a larger project, several additional measures were taken during the MRI session.

### 2.4 Neuroimaging methods

#### 2.4.1 Neuroimaging Methods: MRI Data Acquisition

We acquired MRI data using a 3T MRI scanner (Philips Achieva TX, Erlangen, Germany) with a standard whole-head coil. For functional MRI scans, T2*-weighted gradient echo-planar images were collected (repetition time = 2.2 sec, echo time = 30 msec, flip angle = 8 degrees, sequential acquisition: 38 slices, voxel size = 2.75 × 2.75 × 2.75 mm, 80 × 80 matrix, field of view = 220 × 220 × 115 mm). Functional scans were acquired during three runs (corresponding to the three task blocks), which consisted of 150 dynamic scans each. Prior to the first functional scan of each run, we acquired five dummy scans. Stimuli were displayed on a screen that participants could view through a mirror attached to the head coil. Participants’ head movements were restricted by using foam inserts at one or both sides of the head. In addition to the fMRI sequences, we collected structural images for anatomical reference (duration of 4 min and 12 s, high resolution 3D T1, repetition time = 7.9 ms, echo time = 3.5 ms, flip angle = 8 degrees, 3D matrix size for 3D acquisitions: 228 × 177 × 155 slices, axial slice orientation, voxel size = 1.1 × 1.1 × 1.1 mm, field of view = 250 × 196 × 170 mm). T1 dummy scans for stabilization were automatically discarded by the scanner.

#### 2.4.2 Neuroimaging methods: preprocessing

Data were analyzed using SPM12 (Welcome Department of Cognitive Neurology, London, United Kingdom) using the following steps: realignment, slice-time correction, spatial normalization to T1 templates, spatial smoothing with a 6-mm FWHM isotropic Gaussian kernel. Subsequently, all volumes were resampled to voxels of 3x3x3 millimeters. Our templates were based on the MNI305 stereotaxic space (Cocosco et al., 1997).

#### 2.4.3 Neuroimaging methods: first level analyses

To perform first-level individual analyses, we used the general linear model in SPM12. We modelled the fMRI time series as a series of two events convolved with the hemodynamic response function (HRF). More specifically, we first modelled social feedback onset with a zero duration and with separate regressors for the feedback conditions (i.e., Positive, Negative, Neutral). Second, we modelled the noise blast start for the length of the noise blast duration, with separate regressors for noise blasts following Positive, Negative, and Neutral feedback. Each run was modeled as a separate block. In addition, six motion parameters were included as nuisance regressors. Invalid trials (on which participants failed to respond, 1.72% of trials) were modeled separately as a covariate of no interest and were excluded from further analyses. Least-square parameter estimates of the height of the best-fitting canonical hemodynamic response function were used for each condition in pairwise contrasts. These pairwise comparisons led to participant-specific contrast images, which were subsequently submitted to second-level group analyses.

#### 2.4.4 Neuroimaging methods: second level analyses

We first performed a full factorial analysis of variance (ANOVA) with three levels (Positive, Negative, and Neutral feedback) to examine the neural responses to social feedback on a whole-brain level. More specifically, we calculated and tested the contrasts “Positive vs. Negative valence,” “Positive vs. Neutral valence,” “Negative vs. Neutral valence” (and the reversed contrasts) to investigate which brain regions that were specifically activated for social rejection or social acceptance. In addition, we calculated the conjunction “(Positive + Negative) vs. Neutral valence” (and the reversed contrast) to examine which brain regions were specifically activated in response to valenced evaluations.

Second, we exploratively performed another full factorial ANOVA three levels (Positive, Negative, and Neutral feedback) to examine the neural responses during the noise blast on a whole-brain level, using the contrasts “Positive vs. Negative Noise Blast”, “Positive vs. Neutral Noise Blast,” “Negative vs. Neutral Noise Blast”, “(Positive + Negative) vs. Neutral Noise Blast”, (and the reversed contrasts). Finally, we also explored whether brain activity during the noise blast event following positive feedback was associated with the noise blast duration after positive feedback (relative to negative feedback), using a whole brain regression analysis, using the contrasts “Positive vs. Negative Noise Blast,”and “Negative vs. Positive Noise Blast”. All results were corrected using a FDR cluster-corrected threshold of p < .001. Coordinates for local maxima are reported in MNI space. Unthresholded statistical maps of all reported whole-brain analyses are available on Neurovault (Gorgolewski et al., 2015); see https://neurovault.org/collections/THUHIXAC/.

#### 2.4.5 Neuroimaging Methods: Region-of-Interest analyses (ROIs).

To test for neural differences related to social feedback evaluation, we created 4 ROIs using the MarsBaR toolbox (Brett, Anton, Valabregue, and Poline, 2002) for SPM12 for which we extracted parameter estimates for the left Insula (coordinates x = −36, y = 23, z = -2), right Insula (x = 33, y = 20, z = −11), ACC (x = 0, y = 38, z = 16) (Achterberg et al., 2016), and dlPFC (x = 48, y = 17, z = 37) (Achterberg et al., 2018, van de Groep et al., 2021), based on a-priori hypotheses. All ROIs were created by extracting 10 mm spheres around the specified coordinates. For the 4 a-priori defined ROIs, we applied Bonferroni correction for correlated variables with a threshold of α = 0.0287 (Perneger, 1998). A more detailed description of ROI analyses can be found in the supplement.

### 2.5 Statistical analyses

We followed all analyses steps as detailed in our pre-registration on the Open Science Framework (https://osf.io/d6fku/). Behavioral and ROI data were analyzed using R (Version 4.0.1, R Core team, 2020). Prior to analyses, assumptions were checked. We identified two univariate noise blast duration outliers for positive feedback. These univariate outlier scores were winsorized (Tabachnick and Fidell, 2013). Results did not change before and after winsorizing. Here, we report the winsorized results.

## 3 Results

### 3.1 Behavioral results

#### 3.1.1 Behavioral results: Social feedback × Group

**Fig. 2:**
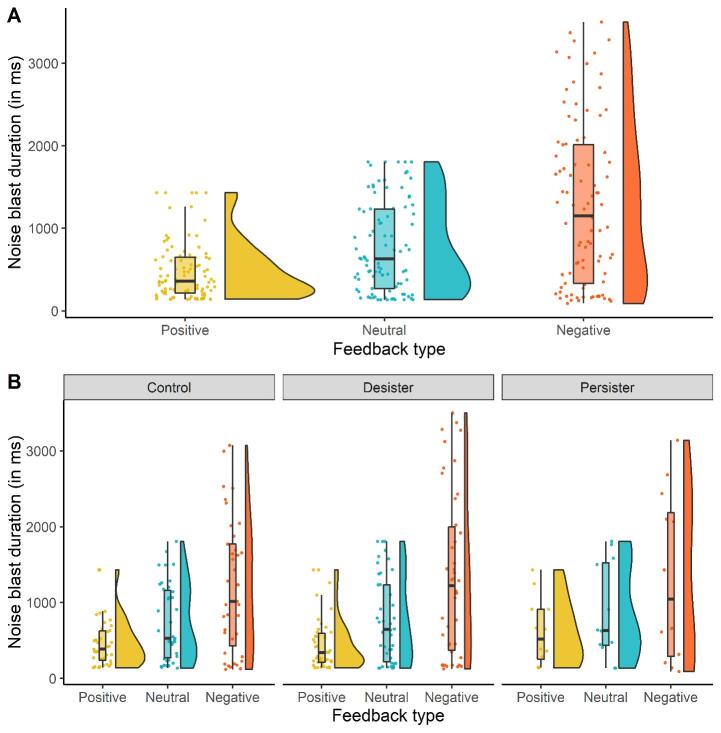
(A) Average noise blast duration following social feedback during the SNAT. Noiseblast duration was longest following Negative feedback, shorter for Neutral feedback and shortest for Positive feedback. (B) Average noise blast duration following social feedback in the different groups (Persisters, Desisters and Controls).

To test whether social feedback and Group status interactively influenced noise blast duration, we performed a repeated-measures ANOVA with Feedback type (Positive vs. Neutral vs. Negative) and Group (Persister vs. Desister vs. Control) as independent variables, and noise blast duration as dependent variable. As can be seen in Fig. 2A, there was a main effect of Feedback type, *F* (1.18, 104.26) = 44.45, *p* < .001, $\eta_{p}^{2}$ = 0.34, indicating that noise blasts were longest following negative feedback (*M* = 1274.04, *SD* = 992.45), shorter for neutral feedback (*M* = 759.86, *SD* = 542.18), and shortest for positive feedback (*M* = 476.93, *SD* = 346.42; all post hoc comparisons (Bonferroni-corrected), *p*’s < 0.001), in line with hypothesis 1a. We found no main effect of Group, nor an interaction effect between Feedback type and Group, all *p*’s > 0.05 (Fig. 2B). Hence, in line with this omnibus test and contrary to our hypothesis 1b, we did not find differences in noise blast duration following social rejection (i.e., negative social feedback) between persisters and desisters, or persisters and controls, all *p*’s > 0.05.

#### 3.1.2 Behavioral results: social feedback × psychopathic traits

**Fig. 3:**
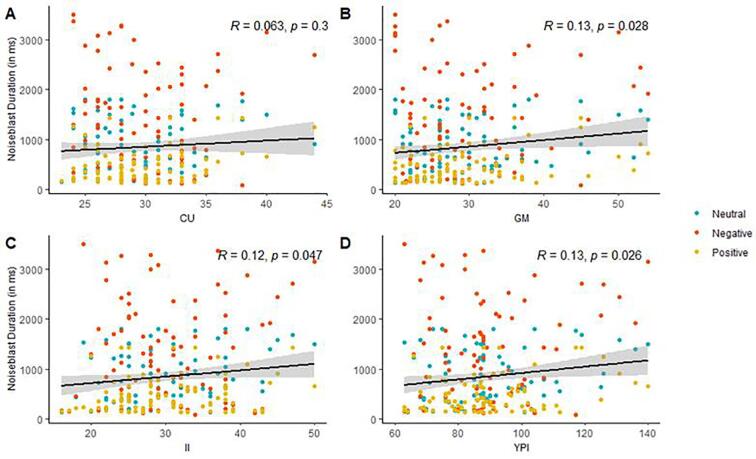
(A-C) Association between Callous-Unemotional (CU), Grandiose-Manipulative (GM) and Impulsive-Irresponsible (II) traits and noise blast duration. (D) Association between Total psychopathic traits (YPI) scores and noise blast duration.

To investigate whether psychopathic traits influence noise blast duration (hypothesis 1c), we performed repeated measures ANOVAs with Feedback type and Psychopathic traits as independent variables, separately for each trait dimension and the total YPI score (Callous-Unemotional, Grandiose-Manipulative, Impulsive-Irresponsible, YPI Total). Visual inspection and correlation analysis of the association between Grandiose-Manipulative and Impulsive-Irresponsible traits and noise blast duration indicated a positive association, implying that higher Grandiose-Manipulative traits (*R* = 0.13, *p* = .028), and higher Impulsive-Irresponsible traits (*R* = 0.12, *p* = .047) are associated with longer noise blast durations (see Fig. 3B-C). However, it should be noted that these associations did not survive corrections for multiple testing. Likewise, the ANOVA with psychopathic traits and Feedback type as independent variables did not reveal a significant main effect of Grandiose-Manipulative traits, *F* (1, 91) = 3.03, *p* = .085, $\eta_{p}^{2}$ = 0.032, Callous-Unemotional traits, *F*(1, 91) = 0.68, *p* = .41, $\eta_{p}^{2}$ = 0.07, or Impulsive-Irresponsible traits, *F*(1, 91) = 2.48, *p* = .19, $\eta_{p}^{2}$ = 0.03 on noise blast duration, nor any interactions between feedback type and the three trait dimensions, all *p*’s > 0.68. Also for the total YPI score, visual inspection and correlation analysis revealed a positive correlation (*R* = 0.13, *p* = .026, see Fig. 3D), but the ANOVA with YPI score and Feedback type as independent variables revealed no significant main effect of YPI score, *F*(1, 91) = 3.13, *p* = .08, $\eta_{p}^{2}$ = 0.03, nor interaction effect between feedback type and total YPI score, *F*(1.19, 108.08) = 0.05, *p* > .87.

### 3.2 Neural results feedback processing

#### 3.2.1 Confirmatory whole brain analysis

**Fig. 4:**
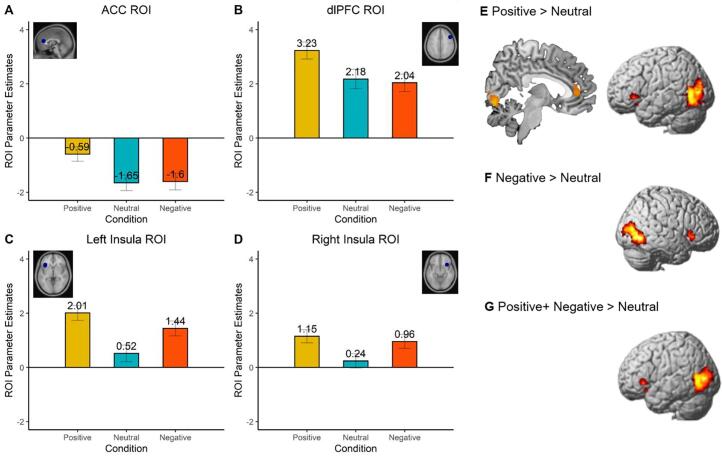
(A-D) Task condition effects (for social feedback) in three pre-defined ROIs (ACC, left and right Insula) and one exploratory ROI (dlPFC). In general, activation was highest for Positive feedback than for Negative and Neutral feedback. (E-G) Whole brain full factorial ANOVA conducted at the group level for the contrasts Positive vs. Neutral feedback (E), Negative vs. Neutral feedback (F) and Positive + Negative vs. Neutral (G).

To examine neural responses on the whole brain level, we performed a whole brain full-factorial ANOVA with Feedback type (Negative, Positive, Neutral) as within-subject factor (see Table 2 for an overview of the results). First, the “Positive > Neutral” feedback contrast resulted in significant activation in the right ACC / mPFC and left Insula / IFG (Fig. 4E). Second, the Valence “(Positive + Negative) vs. Neutral” contrast showed significant activity in the left Insula / IFG (Fig. 4G). Third, the “Negative > Neutral” Feedback contrast yielded significant activation in the right Insula / IFG (Fig. 4F). Together, these results indicate increased brain activation in the left and right Insula and Anterior Cingulate Cortex (ACC) following positive and/or negative feedback, when compared to neutral feedback (consistent with hypothesis 2a).

**Table 2:** MNI coordinates of local maxima activated the contrasts (1) “Positive > Negative”, (2) “Positive > Neutral”, (3) “ (Positive + Negative) > Neutral”, (4) “Positive > (Negative + Neutral)”, (5) “(Positive + Neutral) > Negative”, (6) “Negative > Neutral”, (7) “Negative > (Positive + Neutral)”, and (8) “Neutral > Negative” during the feedback event for the Social Network Aggression Task. Results were FDR cluster-corrected using p < 0.001.The reversed contrasts “Negative > Positive”, “Neutral > Positive” and “(Negative + Neutral) > Positive”, “Neutral > (Positive + Negative) did not result in significant effects. See https://neurovault.org/collections/THUHIXAC/ for a full, unthresholded overview of activation.

Area of activation	MNI Coordinates			Test statistic	Cluster Size
	x	y	z	*T*	
*Positive > Negative feedback*					
Lingual_R	4	−78	−8	7.90	3591
Angular_R	40	−70	42	5.68	755
Frontal_Mid_R	30	22	52	5.64	848
Putamen_L	−28	4	−2	5.11	730
Cingulum_Post_L	0	−34	24	4.95	798
Frontal_Inf_Oper_L	−44	6	26	4.87	437
Frontal_Inf_Tri_L	−46	30	20	4.70	341
Frontal_mid_L	−28	10	54	4.46	472
Parietal_Inf_L	−34	−74	44	4.46	207
Precuneus_R	4	−68	48	4.39	213
Parietal_Inf_L	−50	−36	48	4.30	242
Supp_Motor_Area_L	−6	−10	52	4.19	215
*Positive > Neutral feedback*					
Fusiform_R	24	−74	−10	7.62	4841
Frontal_Inf_Tri_L	−34	28	2	5.93	541
Cingulum_Ant_R	6	42	4	4.09	233
*Positive + Negative > Neutral feedback*					
Temporal_Inf_R	48	−66	−6	7.8	2638
Occipital_Mid_L	−42	−80	6	7.28	1609
Frontal_Inf_Tri_L	−36	28	2	5.90	335
*Positive > Negative + Neutral feedback*					
Lingual_L	−20	−76	−10	8.29	3038
Insula_L	−30	18	6	5.43	857
Occipital_Mid_L	−28	−82	20	5.19	239
Cingulum_Post_L	−2	−36	24	5.11	421
Supp_Motor_Area_R	4	6	48	4.36	383
Parietal_Inf_R	52	−56	40	4.26	186
Frontal_Inf_Oper_L	−44	6	26	4.14	176
Frontal_Inf_Tri_L	−40	44	12	4.07	184
Cingulum_Ant_R	10	42	10	3.85	204
*Positive + Neutral > Negative feedback*					
Frontal_Mid_R	30	2	52	5.99	857
Lingual_R	4	−80	−4	5.79	2370
Angular_R	40	−72	40	5.77	529
Frontal_Mid_L	−28	10	54	5.49	436
Cingulum_Mid_R	4	−38	38	5.07	358
Frontal_Inf_Tri_L	−46	30	20	4.60	204
Parietal_Inf_L	−34	−74	44	4.50	168
Frontal_Inf_Oper_L	−44	4	24	4.49	310
*Negative > Neutral feedback*					
Occipital_Sup_L	−12	−96	8	7.67	1041
Temporal_Inf_R	48	−66	−6	7.11	1508
Frontal_Inf_Orb_R	44	26	−1	4.53	172
*Negative > Positive + Neutral feedback*					
Occipital_Sup_L	−12	−96	8	8.87	165
Calcarine_R	10	−94	10	5.59	326
Occipital_Mid_L	−46	−78	8	5.31	171
Temporal_Inf_R	46	−66	−8	4.51	176
*Neutral > Negative feedback*					
Frontal_Mid_R	30	12	56	4.99	433

Note: Names were based on the aal toolbox in SPM. For functional regions discussed throughout the paper, both the aal label and functional label (between brackets) are displayed.

#### 3.2.2 Confirmatory ROI analyses

##### 3.2.2.1 Confirmatory ROI Analyses: The effect of feedback type (Salience) on Insula and ACC activity

To test whether receiving positive or negative feedback (compared to neutral feedback) resulted in increased brain activation in the Insula and ACC, we also performed repeated measures ANOVAs for the three a priori defined ROIs based on the Achterberg et al. (2016); ACC, left Insula and right Insula (Fig. 4A-D). The analyses resulted in main effects of Feedback type on ACC activation, *F* (2, 176) = 4.39, *p* = .013, $\eta_{p}^{2}$ = 0.048, the left Insula activation, *F* (2, 176) = 6.91, *p* = .001, $\eta_{p}^{2}$ = 0.073, and the right Insula, *F* (2, 176) = 3.54, *p* = .031, $\eta_{p}^{2}$ = 0.039, although the latter did not survive Bonferroni correction. As can be seen in Fig. 4A-D, for all a priori ROIs, activation was highest for positive feedback, and lowest for neutral feedback. Post-hoc tests (Bonferroni-corrected) yielded increased activation in the ACC following positive feedback compared to neutral feedback (*p* = .017). In addition, we observed significant higher activity in the left Insula, *p* = .002, following positive feedback vs. neutral feedback. The other differences between conditions in the ACC, left and right Insula were not significant, all other *p*’s > 0.036 (see supplementary materials Table S2).

##### 3.2.2.2 Confirmatory ROI analyses: the interactive effects of feedback type × Group (Salience) on Insula and ACC activity

**Fig. 5:**
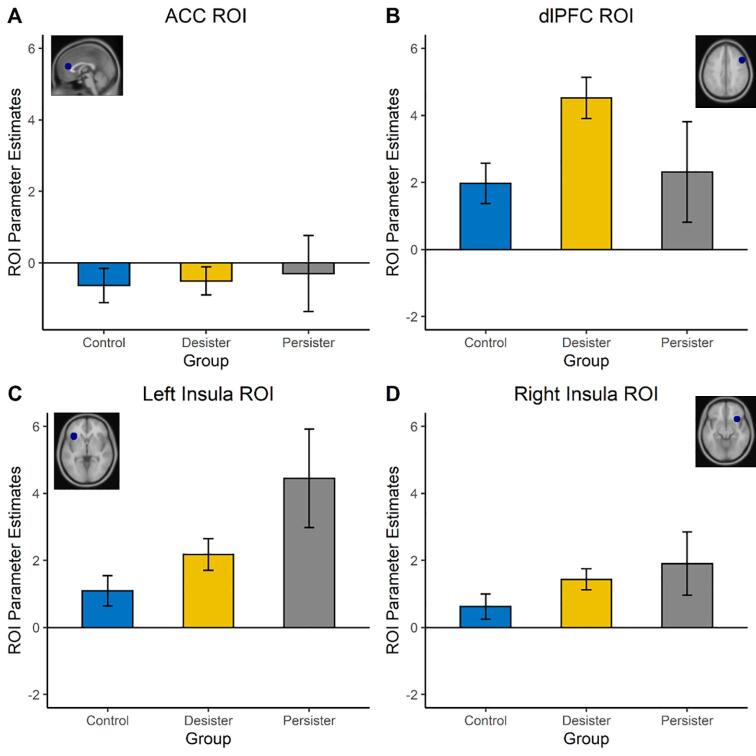
Group effects for three pre-defined ROIs (ACC, left and right Insula) and one exploratory ROI (dlPFC) during feedback processing. For the left Insula (B), right Insula (C), and dlPFC (D), there was a main effect of Group.

To test whether the aforementioned saliency effects in the ACC and Insula would be stronger in persisters when compared to desisters and controls, we performed repeated measures ANOVAs with Feedback type and Group status as independent variables (see Fig. 5). For the left Insula, we observed a main effect of Group, *F* (2, 85) = 3.42, *p* = .037, $\eta_{p}^{2}$ = 0.074, although this effect did not survive Bonferroni correction. For the right Insula, we also found a main effect of Group, *F* (2, 85) = 7.37, *p* = .001, $\eta_{p}^{2}$ = 0.148. Post hoc tests (Bonferroni-corrected) showed a difference in right Insula activation between persisters and controls, *p* = .031, and a significant difference between desisters and controls, *p* = .002 with more activation in persisters and desisters than controls. There was no main effect of Group for the ACC, *F* (2, 85) = 1.46, *p* = .238, and no Feedback × Group interactions for the ACC, *F* (3.87, 164.34) = 0.55, *p* = .69, left Insula, *F* (3.89, 165.41) = 1.14, *p* = .34, or right Insula, *F* (3.95, 167.98) = 0.62, *p* = .65.

##### 3.2.2.3 Confirmatory ROI Analyses: The interactive effects between feedback type × Psychopathic traits (Salience) on Insula and ACC activity

To investigate whether psychopathic traits influence brain activation in the Insula and ACC, we tested whether the three psychopathic trait dimensions and total scores (i.e., Callous-Unemotional, Grandiose-Manipulative, Impulsive-Irresponsible and YPI Total) influenced saliency difference scores (i.e., parameter estimates for “(Positive + Negative) > Neutral”) for the ACC, left and right Insula. Contrary to our hypothesis 2c, we found no evidence that psychopathic traits differentially influence activity in these areas (see supplementary materials, Table S3).

### 3.3 Confirmatory ROI Analyses: the effect of feedback type on dlPFC activity

The next question was to test whether there were significant correlations between dlPFC activity and noise blast for the contrasts “Negative > Positive” and “Negative > Neutral”. Before testing these associations, we explored whether there were main effects of Group or Group × Feedback type interactions in this ROI.

#### 3.3.1 Confirmatory ROI Analyses: the interactive effect of feedback type × Group status on dlPFC activity

To test whether Feedback type and Group interactively influenced activity in the dlPFC, we performed repeated measures ANOVA with Feedback type and Group as the independent variables, and dlPFC parameter estimates as the dependent variable. This analysis yielded a main effect of Group, *F* (2, 85) = 9.37, *p* < .001, $\eta_{p}^{2}$ = 0.181, see Fig. 5B. Post hoc tests indicated that desisters showed increased dlPFC activity compared to controls, *p* < .001. The other differences between conditions and groups were not significant, all other *p*’s > 0.12.

#### 3.3.2 Confirmatory brain behavior associations: dlPFC activity during feedback processing following negative feedback

Contrary to our expectations (hypothesis 3a), there were no significant correlations between dlPFC activity following negative feedback and noise blast duration following negative feedback (relative to positive and neutral feedback), all *p*’s > 0.57 (corrected for multiple-testing).

### 3.4 3 Confirmatory brain behavior associations: dlPFC activity during feedback processing following negative feedback between groups

To test for differences between groups with regard to the observed associations between dlPFC activity and noise blast duration following negative feedback (compared to both neutral and positive feedback), we computed fisher r-to-z transformations. Subsequently, we tested whether the correlations were significantly different between groups (i.e., control vs. persisters, control vs. desisters, and persisters vs. desisters) (Lenhard and Lenhard, 2014), corrected for multiple-testing. Contrary to our hypothesis (3c), group status did not influence associations between dlPFC activity following negative feedback and noise blast duration following negative feedback (relative to both positive and neutral feedback). Hence, contrary to what we expected, there were no differences between persisters and controls, *z* = -0.65, *p* = .26 (Negative vs. Positive), *z* = −1.03, *p* = .15 (Negative vs. Neutral), nor between persisters and desisters, *z* = -0.35, *p* = .36 (Negative vs. Positive), *z* = 1.64, *p* = .051 (Negative vs. Neutral).

### 3.5 Neural results aggressive responses

#### 3.5.1 Exploratory whole brain analyses: neural activity during the noise blast

**Fig. 6:**
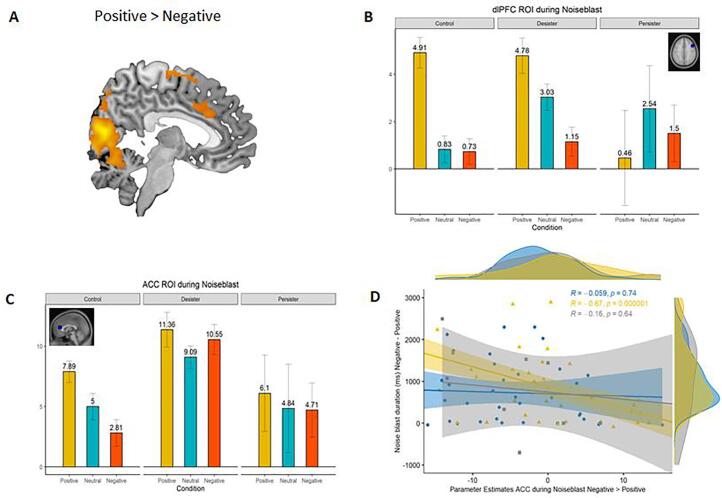
(A) Whole brain regression conducted at the group level for the contrasts Positive vs. Negative feedback. (B) dlPFC parameter estimates during the noise blast event. There was a significant interaction effect between condition and group. (C) ACC parameter estimates during the noise blast event. (D) Difference scores in ACC activity (Negative > Positive feedback) and noise blast duration (Negative > Positive feedback).

Based on prior findings, we explored neural activity during the noise blast event (van de Groep et al., 2021). Several contrasts showed significant differences in activation during the whole brain analyses during the noise blast event (see Table 3). First, the contrast “Positive > Negative Noise Blast” resulted in more activity in the left IFG, right frontal middle gyrus and left putamen (see Fig. 6A). Second, the contrast “Positive > Neutral Noise Blast” resulted in increased activity in the right Calcarine, left Supramarginal gyrus and right Angular gyrus. Finally, the “Positive > (Negative + Neutral) Noise Blast” contrast resulted in more activity in the left IFG, left Cerebellum, left Supramarginal gyrus, left Fusiform gyrus and right Frontal middle gyrus.

**Table 3:** MNI coordinates of local maxima activated the contrasts (1) “Positive > Negative”, (2) “Positive > Neutral” and (3) “Positive > (Negative + Neutral) during the noise blast event for the Social Network Aggression Task. Results were FDR cluster-corrected using p < 0.001. The reversed contrasts did not result in significant effects. See https://neurovault.org/collections/THUHIXAC/ for a full, unthresholded overview of activation.

Area of activation	MNI Coordinates			Test statistic	Cluster Size
	x	y	z	*T*	
*Positive > Negative (during Noise Blast)*					
Occipital_Sup_L	−14	−78	−2	6.56	12,184
Frontal_Inf_Oper_L	−56	4	24	5.61	4404
Frontal_Mid_R	42	6	54	5.05	968
Frontal_Inf_Oper_L	−40	28	34	4.79	793
Putamen_L	−38	−16	10	4.11	324
*Positive > Neutral (during Noise Blast)*					
Calcarine_R	−2	70	8	5.47	2859
SupraMarginal_L	−42	−50	36	4.64	509
Angular_R	46	−58	46	4.44	466
*Positive > Negative + Neutral (during Noise Blast)*					
Cerebelum_6_L	−10	−76	−2	6.45	9551
SupraMarginal_L	−44	−52	40	5.23	2711
Frontal_Mid_R	42	6	54	5.10	1247
Fusiform_L	−46	−66	−8	5.01	307
Frontal_Inf_Oper_L	−40	30	34	4.61	495

Note: Names were based on the aal toolbox in SPM. For functional regions discussed throughout the paper, both the aal label and functional label (between brackets) are displayed.

#### 3.5.2 Exploratory ROI Analyses: dlPFC activity during the noise blast

Based on earlier findings that suggest differential reactivity to social feedback of the dlPFC during the noise blast event (van de Groep et al., 2021), we extracted ROI values from the whole brain analysis, (coordinates: x = -34, y = 36, z = 16; using a 10 mm sphere) to examine this possibility in more detail, and explore whether this reactivity differed between groups, using a Feedback type × Group ANOVA. We found a main effect of Feedback type, *F*(1.81, 153.66) = 4.74, *p* = .012, $\eta_{p}^{2}$ = 0.053. Post hoc tests revealed significantly higher dlPFC activity during noise blast responses following positive feedback compared to negative feedback, *p* < .001, and neutral feedback, *p* = .008. In addition, we observed a Feedback type × Group interaction (See Fig. 6B), *F*(3.62, 153.66) = 3.11, *p* = .021, $\eta_{p}^{2}$ = 0.068, which indicated that the persister group showed less dlPFC activity during the noise blast event following positive feedback compared to controls, *p* = .02, and desisters, *p* = .02. Follow-up ANOVAs also revealed that persisters did not respond differently to the different feedback types, *F* (2, 20) = 0.38, *p* = 696, $\eta_{p}^{2}$ = 0.004, unlike the controls, *F* (2, 68) = 16.51, *p* < .001, $\eta_{p}^{2}$ = 0.327, and desisters, *F* (2, 82) = 8.03, *p* < .001, $\eta_{p}^{2}$ = 0.163. The latter two groups both showed most activity following positive feedback, less after neutral feedback, and least after negative feedback.

#### 3.5.3 Exploratory whole brain regression: activity during the noise blast

Finally, we explored whether brain activity during the noise blast event following positive feedback was associated with the noise blast duration after positive feedback (relative to negative and neutral feedback), using a whole brain regression analysis. We found increased activity following positive feedback was associated with shorter noise blast duration in several areas (see Table 4 and Fig. 6C), including the ACC and Dorsal Striatum (Caudate and Putamen). Visual inspection revealed that these associations were mainly driven by the desister subgroup. To further explore this effect, we examined whether brain-behavior associations for these areas differed between groups, using the same approach as described in the previous section. These analyses revealed that desisters showed stronger negative associations in the ACC compared to the controls (see Fig. 6D), *z* = 5.037, *p* < .001, and persisters, *z* = 2.543, *p* = .005, as well as in the left Caudate, compared to controls, *z* = 4.116, *p* < .001, and persisters, *z* = 2.572, *p* = .005. There were no significant differences between persisters and controls.

**Table 4:** MNI coordinates of local maxima activated the contrast “Negative > Positive” for the Whole brain regression Social Network Aggression Task. Results were FDR cluster-corrected using p < 0.001. The reversed contrasts “Positive > Negative” did not result in significant effects. See https://neurovault.org/collections/THUHIXAC/ for a full, unthresholded overview of activation.

Area of activation	MNI Coordinates			Test statistic	Cluster Size
	x	y	z	*T*	
*Negative > Positive During Noiseblast*					
Cuneus_L	−12	−86	2	7.66	9647
Putamen_R	24	−10	2	5.01	242
Parietal_Sup_R	50	–32	54	4.81	232
Supp_Motor_Area_R	0	−4	68	4.75	232
Cingulum_Ant_R	−6	30	26	4.67	371
Caudate_L	−10	4	4	4.37	221

Note: Names were based on the aal toolbox in SPM. For functional regions discussed throughout the paper, both the aal label and functional label (between brackets) are displayed.

Note: Names were based on the aal toolbox in SPM. For functional regions discussed throughout the paper, both the aal label and functional label (between brackets) are displayed.

## 4 Discussion

An important developmental question concerns why some people who show antisocial behavior in childhood persist in antisocial behavior into early adulthood, whereas others desist from this trajectory (Hyde et al., 2018, Laub and Sampson, 2021). In this study, we addressed this question using a social aggression paradigm to examine behavioral and neural responses to social feedback in young adults with and without a history of antisocial behavior. We examined the role of social context in retaliatory aggressive behavior using two different, but complementary approaches: a developmental group trajectory approach (i.e., comparing desisters/persisters/controls) and an individual differences approach by examining the association with psychopathic traits. We showed three important behavioral and neural development findings. First, when participants received rejection relative to neutral and positive feedback, they showed higher retaliatory aggression (noise blasts), regardless of group. Moreover, higher retaliatory aggression responses were associated with higher levels of psychopathic traits. Second, when receiving social feedback, individuals with persistent or desistent trajectory of antisocial behavior showed dissociable patterns of neural activity; with higher activity in the Insula for the desisting and persisting trajectory groups (compared to controls) and higher activity in dlPFC only for the desisting trajectory group (compared to the persistent and control groups). Third, when administering the noise blast, participants in the desister and control groups showed increased activity in dlPFC and ACC for positive relative to neutral and negative feedback, whereas ACC activity correlated most strongly with inhibiting noise blasts in the desisting trajectory group. Together, these findings provide novel insights in similar and dissociable patterns of brain activity that suggest differences between various subgroups in how people process social information, and preliminary insights in whether and how they adapt their behavior accordingly in social situations during development.

Research on antisocial behavior is building an increasingly detailed picture of the etiology and maintenance of aggressive behavior throughout development (Moffitt, 2018). Although aggressive behavior in social contexts has been well characterized in childhood and adolescence (Achterberg et al., 2017, Achterberg et al., 2018, Achterberg et al., 2020, Bertsch et al., 2020), far less is known about such behavior in early adulthood, particularly in high-risk groups (Bertsch et al., 2020) – even though this developmental period may be a crucial period for the (dis)continuity of antisocial behavior (Hyde et al., 2018, Monahan et al., 2009). This study used a social aggression paradigm that combined social feedback with the possibility to retaliate by pressing a noise blast (Achterberg et al., 2016, Chester et al., 2014). As expected, noise blasts were longer following rejection feedback, shorter for neutral and shortest for positive feedback, replicating prior findings (Achterberg et al., 2016). Interestingly, this pattern was not different between the persisting, desisting and control subgroups, showing that the basic retaliation response is observed in participants with and without a history of antisocial behavior. Yet, the overall noise blast duration correlated with individual differences in psychopathic traits. More specifically, Grandiose-Manipulative and Impulsive-Irresponsible traits, as well as the total YPI score, were positively associated with noise blast duration. These findings are consistent with prior studies showing positive associations between (subdimensions of) psychopathic traits and reactive aggression (Blais et al., 2014). This pattern also speaks to prior studies noting that a dimensional approach can provide a more sensitive index compared to a categorical approach of DSM diagnoses (Garvey et al., 2016). However, it should be noted that these associations between psychopathic traits and reactive aggression were small in size, and no longer significant when correcting for multiple testing, or when social feedback was added to the ANOVA model, signaling that future research is warranted to better understand how social context influences the link between psychopathy and aggression (Brennan et al., 2018, van Baardewijk et al., 2009). In addition, the similarity in behavioral patterns between groups raises the question whether future studies should employ stronger social context manipulations that result in more pronounced differences between groups.

Examining the neural basis of information processing can provide a better understanding of underlying neural responses that cannot always be observed at the level of behavior. Indeed, this study replicated the neural pattern observed in prior studies showing that feedback that signals acceptance or rejection leads to increased activity in the Insula and ACC (Dalgleish et al., 2017), possibly indicating higher saliency for feedback that has valence information (Dalgleish et al., 2017, Eisenberger et al., 2011), or increased monitoring of such socially salient cues, which facilitates updating and selecting appropriate action plans (Puiu et al., 2020). As predicted, we observed that the subgroups differed in neural responses to feedback, although this was observed at the level of general feedback processing and was not valence specific. That is, individuals with a persisting and desisting antisocial trajectory showed higher activity overall in the Insula to all types of social feedback, relative to the control group. Possibly, this exaggerated activity indicates increased salience of social cues in these groups, and/or increased allocation of processing resources to self-relevant and motivational social information (Baskin-Sommers and Newman, 2014, Perini et al., 2018, Puiu et al., 2020). These findings fit with earlier studies showing that antisocial behavior is associated with altered anterior Insula function and structure (Dugré et al., 2020, Noordermeer et al., 2016). However, evidence on the direction of this alteration is currently inconclusive, given that other functional studies tend to find anterior Insula hypoactivity during emotional processing in antisocial populations (Dugré et al., 2020), rather than hyperresponsiveness. Possibly, the direction of these anterior Insula effects may be context-dependent, contingent on whether the social cues are self-relevant and require a behavioral response (Perini et al., 2018). Our finding that increased anterior Insula activity during social feedback processing seems specific to individuals with a history of antisocial behavior also raises the question whether this neural sensitivity is already apparent early in development, whether it arises as a consequence of repeated antisocial behavior, repeated negative social interactions, or a combination. As such, future research should investigate when and how environmental factors and social interactions shape neural sensitivity to social feedback in populations who display early-onset antisocial behavior during different developmental stages (Ellis et al., 2011, Foulkes and Blakemore, 2018, Muscatello et al., 2020, Schriber and Guyer, 2016).

A novel finding that was not predicted in the pre-registration was that the individuals with a desisting trajectory recruited the dlPFC more strongly during general feedback processing, relative to control and persisting subgroups. Possibly, this increased activity in the desister group reflects increased attention to changing task demands (i.e., to context-dependent changes in feedback presentation between trials), which supports subsequent top-down cognitive control or emotion regulation by preparing response maintenance, selection or inhibition (Niendam et al., 2012). In line with this idea, dlPFC activity during feedback processing in the desister group was highest during positive feedback, compared to neural and negative feedback. Together with the notion of structural and functional dlPFC impairments in antisocial populations (Yang and Raine, 2009), our finding suggests that increased dlPFC activity may play a role in desisters’ ability to successfully adapt their responses and refrain from aggression and other forms of antisocial behavior. However, as of yet, it remains unclear whether this increased dlPFC activity underlies successful behavioral adaptation itself, or reflects increased effortful control which is initiated by desisters after learning that aggressive, retaliatory behavior may not be an optimal, socially adaptive strategy. Future studies may employ transcranial magnetic stimulation (TMS) to further test whether the dlPFC is indeed causally involved in behavioral adaptation, and whether altering dlPFC function in individuals with persistent antisocial behaviors may help them to successfully adapt their behavior.

A final exploratory focus concerned the neural correlates of delivering the noise blast following positive, neutral and negative feedback. Direct comparisons revealed increased activity in the dlPFC and ACC specifically for positive feedback (in the desister and control groups, but not in the persister group) which is the condition where the participants gave the shortest noise blasts. This observation led to the hypothesis that these regions may be involved in the inhibition of retaliation following positive feedback (Brockett et al., 2020, Crew et al., 2021, van Heukelum et al., 2021). Whole brain regression analyses confirmed that successfully being able to regulate aggression after social acceptance was associated with increased activity in the ACC and dorsal striatum (caudate and putamen) during retaliatory responses. Our findings fit with earlier studies showing a negative association between retaliation and ACC activity (Alegria et al., 2016, Gavita et al., 2012, Krämer et al., 2007, Yang and Raine, 2009) and dorsal striatal activity during retaliatory responses (Kose et al., 2015, Krämer et al., 2007, Lotze et al., 2007), corroborating that these areas are important for adaptive behavioral control of retaliatory responses in a social context (Brockett et al., 2020, Crew et al., 2021, van Heukelum et al., 2021, Bertsch et al., 2020, Grahn et al., 2008). Moreover, the cluster we identified in the ACC was also similar to the dorsal-frontomedial cortex area that has been implicated in the voluntary, intentional inhibition of actions (Filevich et al., 2012), which was confirmed through visual inspection. Interestingly, our findings further revealed the negative association between aggression following positive feedback and activity in the ACC and dorsal striatum during retaliation was stronger in desisters, compared to controls and persisters. Together, these findings point towards a possible adaptive behavioral control mechanism that enables adolescents to desist from antisocial behavior in early adulthood (Bersani and Doherty, 2018, Bertsch et al., 2020, Krämer et al., 2007, Moffitt et al., 2002), albeit one that requires significantly more effort to adjust behavior compared to individuals without a history of antisocial behavior.

Finally, our finding that the persistent antisocial behavior group did not show these patterns suggests that they may be less motivated to adapt their behavior, possibly because affiliative and prosocial behavior is not rewarding for them (Foulkes et al., 2014), or they have failed to learn to inhibit aggressive responses, due to problems in stimulus-reinforcement learning and response outcome learning (e.g. cf. Violence Inhibition Mechanism (VIM; Blair, 1995, Blair, 2001, Blair, 2013)). Future research should further examine these possibilities, and investigate whether this is especially true in contexts that lack interpersonal signals that cue distress in others (Blair, 1995, van Baardewijk et al., 2009).

## 5 Limitations and future directions

Although the current study has many strengths, including a relatively large sample with varying risk of severe antisocial behavior, and assessment of social rejection and subsequent aggression within one experimental fMRI paradigm, and a combination of a dimensional and developmental group trajectory approach, the results should be interpreted in the context of limitations. First, in both the antisocial groups (persisters and desisters), some individuals had (other) mental health problems and/or comorbidities (see Table S4 for more details). While such comorbidities are common in the population of interest (Nichita and Buckley, 2020), and our sample is thus representative in that sense, results should be interpreted with this in mind (Rappaport and Barch, 2020, Simmons et al., 2008, Vetter et al., 2018). Likewise, we cannot rule out that our findings might be influenced by other differences in demographic characteristics between groups, such as gender or age. For example, even though all participants were in the developmental stage of early adulthood, participants in the control group were significantly younger than participants in the other groups. Second, the sub-sample of individuals with a persistent history of antisocial behavior was relatively small in size, which may have limited our power to detect significant effects. Third, while our aim was to test differences between people who persisted versus desisted in antisocial behavior regardless of age of onset, we acknowledge that age of onset is an important construct to understand the development of antisocial behavior, which should be taken into account in future studies. Although all participants in the persister and desister groups showed an early onset of antisocial behavior (indicated by the young age at which they committed a reported index crime), our sample (size) is not well suited to fully take this factor and possible differences between early vs. later onset into consideration. Fourth, the reliability of the Callous-Unemotional scales in both samples (controls and childhood arrestee cohort) was poor, which fits with earlier research suggesting that affective dimensions of psychopathy are difficult to accurately assess using self-report measures (Hillege et al., 2010; see also Cardinale and Marsh, 2020). Hence, while our study provides preliminary evidence for links between individual differences and social aggression, future work needs to replicate our findings using different types of assessments (Boonmann et al., 2015). Finally, in the current paper, we did not specifically examine heterogeneity within persisting or desisting developmental trajectories. However, recent studies indicate that while many people who desist from antisocial behavior show a positive social development, this is not always the case (Moffitt et al., 2002). For instance, some people who desist might no longer show antisocial behavior, but nevertheless display abnormal social behavior, that is characterized by social isolation and internalizing problems (Moffitt et al., 2002, Carlisi et al., 2021). Future studies should try to further disentangle heterogenous patterns within persistent and desistent trajectories, and examine how the interplay between neural vulnerabilities and social interactions give rise to diverging patterns of social behavior in early adulthood (Carlisi et al., 2021).

## 6 Conclusion

In conclusion, our study provides new evidence of both similar and dissociable patterns of neural activation in individuals with persisting and desisting antisocial trajectories in brain areas that signal socially salient and self-relevant information (Perini et al., 2018), including the bilateral Insula and ACC, and brain areas that are important for behavioral control, such as the dlPFC (Taber-Thomas and Pérez-Edgar, 2015). Given that early adulthood is characterized by continuous neurodevelopment in brain areas that are important for adaptive social behavior, this study may help to unravel sensitivities that allow us to understand why children and adolescents desist from negative developmental trajectories before they enter adulthood (Bersani and Doherty, 2018, van Goozen and Fairchild, 2008).

## CRediT authorship contribution statement

**Ilse H. van de Groep:** Conceptualization, Formal analysis, Investigation, Writing – original draft, Writing – review & editing, Project administration, Visualization. **Marieke G.N. Bos:** Conceptualization, Writing – review & editing, Supervision, Project administration. **Lucres M.C. Jansen:** Conceptualization, Writing – review & editing, Supervision. **Desana Kocevska:** Writing – review & editing. **Anika Bexkens:** Writing – review & editing. **Moran Cohn:** Writing – review & editing. **Lieke van Domburgh:** Writing – review & editing. **Arne Popma:** Writing – review & editing. **Eveline A. Crone:** Conceptualization, Validation, Resources, Writing – review & editing, Supervision, Funding acquisition.

## Declaration of Competing Interest

The authors declare that they have no known competing financial interests or personal relationships that could have appeared to influence the work reported in this paper.

## References

[b0005] Achterberg M., van Duijvenvoorde A.C.K., van IJzendoorn M.H., Bakermans-Kranenburg M.J., Crone E.A. (2020). Longitudinal changes in DLPFC activation during childhood are related to decreased aggression following social rejection. Proc. Natl. Acad. Sci..

[b0010] Achterberg M., van Duijvenvoorde A.C.K., van der Meulen M., Bakermans-Kranenburg M.J., Crone E.A. (2018). Heritability of aggression following social evaluation in middle childhood: An fMRI study. Hum. Brain Mapp..

[b0015] Achterberg M., van Duijvenvoorde A.C.K., Bakermans-Kranenburg M.J., Crone E.A. (2016). Control your anger! The neural basis of aggression regulation in response to negative social feedback. Soc. Cognitive Affective Neurosci..

[b0020] Achterberg M., van Duijvenvoorde A.C.K., van der Meulen M., Euser S., Bakermans-Kranenburg M.J., Crone E.A. (2017). The neural and behavioral correlates of social evaluation in childhood. Developm. Cognitive Neurosci..

[b0025] Aguilar B., Sroufe L.A., Egeland B., Carlson E. (2000). Distinguishing the early-onset/persistent and adolescence-onset antisocial behavior types: from birth to 16 years. Dev. Psychopathol..

[b0030] Alegria, A. A., Radua, J., Rubia, K. (2016). Meta-Analysis of fMRI Studies of Disruptive Behavior Disorders. Am. J. Psychiatry, 173(11), 1119–1130, 10.1176/appi.ajp.2016.15081089. 10.1176/appi.ajp.2016.15081089 27523497

[b0035] Andershed H.A., Kerr M., Stattin H., Levander S. (2002).

[b0040] Aoki Y., Inokuchi R., Nakao T., Yamasue H. (2014). Neural bases of antisocial behavior: a voxel-based meta-analysis. Social Cogn. Affective Neurosci..

[b0045] Arnett J.J. (2005). The developmental context of substance use in emerging adulthood. J. Drug Issues.

[b0050] Arnett J.J. (2007). Emerging adulthood: what is it, and what is it good for?. Child Develop. Perspectives.

[b0055] Baskin-Sommers A.R., Newman J.P. (2014). Psychopathic and externalizing offenders display dissociable dysfunctions when responding to facial affect. Personality Disorders.

[b0060] Bersani B.E., Doherty E.E. (2018). Desistance from offending in the twenty-first century. Ann. Rev. Criminol..

[b0065] Bertsch K., Florange J., Herpertz S.C. (2020). Understanding brain mechanisms of reactive aggression. Curr. Psychiatry Rep..

[b0070] Bevilacqua L., Hale D., Barker E.D., Viner R. (2018). Conduct problems trajectories and psychosocial outcomes: a systematic review and meta-analysis. Eur. Child Adolesc. Psychiatry.

[b0075] Blair R. (2015). Psychopathic traits from an RDoC perspective. Curr. Opin. Neurobiol..

[b0080] Blair R. (1995). A cognitive developmental approach to morality: Investigating the psychopath. Cognition.

[b0085] Blair R.J.R. (2001). Advances in neuropsychiatry: neurocognitive models of aggression, the antisocial personality disorders, and psychopathy. J. Neurol. Neurosurg. Psychiatry.

[b0090] Blair R.J.R. (2013). The neurobiology of psychopathic traits in youths. Nat. Rev. Neurosci..

[b0095] Blais J., Solodukhin E., Forth A.E. (2014). A meta-analysis exploring the relationship between psychopathy and instrumental versus reactive violence. Criminal Justice Behav..

[b0100] Boonmann C., Jansen L.M.C., ’t Hart-Kerkhoffs L.A., Vahl P., Hillege S.L., Doreleijers T.A.H., Vermeiren R.R.J.M. (2015). Self-reported psychopathic traits in sexually offending juveniles compared with generally offending juveniles and general population youth. Int. J. Offender Therapy Compar. Criminol..

[b0105] Brennan G.M., Crowley M.J., Wu J., Mayes L.C., Baskin-Sommers A.R. (2018). Neural processing of social exclusion in individuals with psychopathic traits: Links to anger and aggression. Psychiatry Res..

[b0110] Brockett A.T., Tennyson S.S., deBettencourt C.A., Gaye F., Roesch M.R. (2020). Anterior cingulate cortex is necessary for adaptation of action plans. Proc. Natl. Acad. Sci..

[b0115] Campbell M.A., Doucette N.L., French S. (2009). Validity and stability of the Youth Psychopathic Traits Inventory in a nonforensic sample of young adults. J. Pers. Assess..

[b0120] Cardinale E.M., Marsh A.A. (2020). The reliability and validity of the inventory of callous-unemotional traits: a meta-analytic review. Assessment.

[b0125] Carlisi C.O., Moffitt T.E., Knodt A.R., Harrington H., Ireland D., Melzer T.R., Poulton R., Ramrakha S., Caspi A., Hariri A.R., Viding E. (2020). Associations between life-course-persistent antisocial behaviour and brain structure in a population-representative longitudinal birth cohort. Lancet Psychiatry.

[b0130] Carlisi C.O., Moffitt T.E., Knodt A.R., Harrington H., Langevin S., Ireland D., Melzer T.R., Poulton R., Ramrakha S., Caspi A., Hariri A.R., Viding E. (2021). Association of subcortical gray-matter volumes with life-course-persistent antisocial behavior in a population-representative longitudinal birth cohort. Dev. Psychopathol..

[b0135] Chester D.S., Eisenberger N.I., Pond R.S., Richman S.B., Bushman B.J., DeWall C.N. (2014). The interactive effect of social pain and executive functioning on aggression: An fMRI experiment. Social Cognit. Affective Neurosci..

[b0140] Cocosco C.A., Kollokian V., Kwan R.-K.-S., Pike G.B., Evans A.C. (1997). BrainWeb: online interface to a 3D MRI simulated brain database. NeuroImage.

[b0145] Cohn M.D., Pape L.E., Schmaal L., van den Brink W., van Wingen G., Vermeiren R.R.J.M., Doreleijers T.A.H., Veltman D.J., Popma A. (2015). Differential relations between juvenile psychopathic traits and resting state network connectivity. Human Brain Mapping.

[b0150] Cohn M.D., Popma A., van den Brink W., Pape L.E., Kindt M., van Domburgh L., Doreleijers T., Veltman D.J. (2013). Fear conditioning, persistence of disruptive behavior and psychopathic traits: an fMRI study. Transl. Psychiatry.

[b0155] Cohn M.D., van Lith K., Kindt M., Pape L.E., Doreleijers T.A.H., van den Brink W., Veltman D.J., Popma A. (2016). Fear extinction, persistent disruptive behavior and psychopathic traits: FMRI in late adolescence. Social Cognit. Affective Neurosci..

[b0160] Cohn M.D., Veltman D.J., Pape L.E., van Lith K., Vermeiren R.R.J.M., van den Brink W., Doreleijers T.A.H., Popma A. (2015). Incentive processing in persistent disruptive behavior and psychopathic traits: a functional magnetic resonance imaging study in adolescents. Biol. Psychiatry.

[b0165] Cohn M.D., Viding E., McCrory E., Pape L., van den Brink W., Doreleijers T.A.H., Veltman D.J., Popma A. (2016). Regional grey matter volume and concentration in at-risk adolescents: Untangling associations with callous-unemotional traits and conduct disorder symptoms. Psychiatry Res.: Neuroimag..

[b0170] Cohn M., van Domburgh L., Vermeiren R., Geluk C., Doreleijers T. (2012). Externalizing psychopathology and persistence of offending in childhood first-time arrestees. Eur. Child Adolesc. Psychiatry.

[b0175] Crew L.A., Covington H.E., Hyman J.M. (2021). Aggression: how the anterior cingulate cortex helps to ensure a fair fight. Curr. Biol..

[b0180] Crone E.A., Dahl R.E. (2012). Understanding adolescence as a period of social–affective engagement and goal flexibility. Nat. Rev. Neurosci..

[b0185] Cyr M., Zheng Y., McMahon R.J. (2020). A long-term look at “early starters”: Predicting adult psychosocial outcomes from childhood conduct problem trajectories. Dev. Psychopathol..

[b0190] Dale A.M. (1999). Optimal experimental design for event-related fMRI. Hum. Brain Mapp..

[b0195] Dalgleish T., Walsh N.D., Mobbs D., Schweizer S., van Harmelen A.-L., Dunn B., Dunn V., Goodyer I., Stretton J. (2017). Social pain and social gain in the adolescent brain: A common neural circuitry underlying both positive and negative social evaluation. Sci. Rep..

[b0200] David C.F., Kistner J.A. (2000). Do positive self-perceptions have a “dark side”? Examination of the link between perceptual bias and aggression. J. Abnorm. Child Psychol..

[b0210] Dugré J.R., Radua J., Carignan-Allard M., Dumais A., Rubia K., Potvin S. (2020). Neurofunctional abnormalities in antisocial spectrum: a meta-analysis of fMRI studies on Five distinct neurocognitive research domains. Neurosci. Biobehav. Rev..

[b0215] Eisenberger N.I., Inagaki T.K., Muscatell K.A., Byrne Haltom K.E., Leary M.R. (2011). The neural sociometer: brain mechanisms underlying state self-esteem. J. Cognit. Neurosci..

[b0220] Ellis B.J., Boyce W.T., Belsky J., Bakermans-Kranenburg M.J., van Ijzendoorn M.H. (2011). Differential susceptibility to the environment: an evolutionary–neurodevelopmental theory. Dev. Psychopathol..

[b0225] Fairchild G., Passamonti L., Hurford G., Hagan C.C., von dem Hagen E.A.H., van Goozen S.H.M., Goodyer I.M., Calder A.J. (2011). Brain structure abnormalities in early-onset and adolescent-onset conduct disorder. Am. J. Psychiatry.

[b0230] Fairchild G., Goozen S.H.M., Calder A.J., Goodyer I.M. (2013). Research Review: Evaluating and reformulating the developmental taxonomic theory of antisocial behaviour. J. Child Psychol. Psychiatry.

[b0240] Filevich E., Kühn S., Haggard P. (2012). Intentional inhibition in human action: the power of ‘no’. Neurosci. Biobehav. Rev..

[b0245] Foulkes L., Blakemore S.-J. (2018). Studying individual differences in human adolescent brain development. Nat. Neurosci..

[b0250] Foulkes L., McCrory E.J., Neumann C.S., Viding E., Ginsberg S.D. (2014). Inverted social reward: associations between psychopathic traits and self-report and experimental measures of social reward. PLoS ONE.

[b0255] Frick P.J., Robertson E.L., Clark J.E., Martel M.M. (2018). Developmental Pathways to Disruptive, Impulse-Control and Conduct Disorders.

[b0260] Garvey M., Avenevoli S., Anderson K. (2016). The national institute of mental health research domain criteria and clinical research in child and adolescent psychiatry. J. Am. Acad. Child Adolesc. Psychiatry.

[b0265] Gavita O.A., Capris D., Bolno J., David D. (2012). Anterior cingulate cortex findings in child disruptive behavior disorders: a meta-analysis. Aggress. Violent Behav..

[b0270] Girard L.-C., Tremblay R.E., Nagin D., Côté S.M. (2019). Development of aggression subtypes from childhood to adolescence: a group-based multi-trajectory modelling perspective. J. Abnorm. Child Psychol..

[b0275] Gorgolewski K.J., Varoquaux G., Rivera G., Schwarz Y., Ghosh S.S., Maumet C., Sochat V.V., Nichols T.E., Poldrack R.A., Poline J.-B., Yarkoni T., Margulies D.S. (2015). NeuroVault.org: A web-based repository for collecting and sharing unthresholded statistical maps of the human brain. Front. Neuroinform..

[b0280] Grahn J.A., Parkinson J.A., Owen A.M. (2008). The cognitive functions of the caudate nucleus. Prog. Neurobiol..

[b0285] Herting M.M., Johnson C., Mills K.L., Vijayakumar N., Dennison M., Liu C., Goddings A.-L., Dahl R.E., Sowell E.R., Whittle S., Allen N.B., Tamnes C.K. (2018). Development of subcortical volumes across adolescence in males and females: a multisample study of longitudinal changes. NeuroImage.

[b0290] Hillege S., Das J., de Ruiter C. (2010). The Youth Psychopathic traits Inventory: Psychometric properties and its relation to substance use and interpersonal style in a Dutch sample of non-referred adolescents. J. Adolescence.

[b0295] Hyde L.W., Waller R., Shaw D.S., Murray L., Forbes E.E. (2018). Deflections from adolescent trajectories of antisocial behavior: contextual and neural moderators of antisocial behavior stability into emerging adulthood. J. Child Psychol. Psychiatry.

[b0305] Insel T., Cuthbert B., Garvey M., Heinssen R., Pine D.S., Quinn K., Sanislow C., Wang P. (2010). Research domain criteria (RDoC): toward a new classification framework for research on mental disorders. Am. J. Psychiatry.

[b0310] Jambroes T., Jansen L.M.C., Ven P.M.V.D., Claassen T., Glennon J.C., Vermeiren R.R.J.M., Doreleijers T.A.H., Popma A. (2018). Dimensions of psychopathy in relation to proactive and reactive aggression: does intelligence matter?. Personality Individ. Differ..

[b0315] Kandel D.B. (1978). Similarity in real-life adolescent friendship pairs. J. Pers. Soc. Psychol..

[b0320] Kose S., Steinberg J.L., Moeller F.G., Gowin J.L., Zuniga E., Kamdar Z.N., Schmitz J.M., Lane S.D. (2015). Neural correlates of impulsive aggressive behavior in subjects with a history of alcohol dependence. Behav. Neurosci..

[b0325] Krämer U.M., Jansma H., Tempelmann C., Münte T.F. (2007). Tit-for-tat: the neural basis of reactive aggression. NeuroImage.

[b0330] Laub, J.H., Sampson, R.J., 2021. 2 Persistence or Desistance? In *Shared Beginnings, Divergent Lives* (pp. 13–35). Harvard University Press. https://www.degruyter.com/document/doi/10.4159/9780674039971-003/html.

[b0335] Lecrubier Y., Sheehan D., Weiller E., Amorim P., Bonora I., Sheehan K.H., Janavs J., Dunbar G. (1997). The mini international neuropsychiatric interview (MINI). A short diagnostic structured interview: Reliability and validity according to the CIDI. Eur. Psychiatry.

[b0340] Lenhard, W., Lenhard, A., 2014. *Testing the Significance of Correlations*. 10.13140/RG.2.1.2954.1367.

[b0345] Lotze M., Veit R., Anders S., Birbaumer N. (2007). Evidence for a different role of the ventral and dorsal medial prefrontal cortex for social reactive aggression: an interactive fMRI study. NeuroImage.

[b0350] Moffitt T.E. (1993). Adolescence-limited and life-course-persistent antisocial behavior: a developmental taxonomy. Psychological review.

[b0355] Moffitt T.E. (2018). Male antisocial behaviour in adolescence and beyond. Nat. Hum. Behav..

[b0360] Moffitt T.E., Caspi A., Harrington H., Milne B.J. (2002). Males on the life-course-persistent and adolescence-limited antisocial pathways: follow-up at age 26 years. Dev. Psychopathol..

[b0365] Monahan K.C., Steinberg L., Cauffman E. (2009). Affiliation with antisocial peers, susceptibility to peer influence, and antisocial behavior during the transition to adulthood. Dev. Psychol..

[b0370] Muscatello M.R.A., Rizzo A., Celebre L., Mento C., Pandolfo G., Cedro C., Battaglia F., Zoccali R.A., Bruno A. (2020). The wounds of childhood: early trauma subtypes, salience and hyperarousal in a sample of adult psychiatric patients. Int. J. Soc. Psychiatry.

[b0375] Neumann C., Pardini D. (2012). Factor structure and construct validity of the self-report psychopathy (SRP) scale and the youth psychopathic traits inventory (YPI) in young men. J. Pers. Disord..

[b0380] Nichita E.C., Buckley P.F., Felthous A.R., Saß H. (2020). The Wiley International Handbook on Psychopathic Disorders and the Law.

[b0385] Niendam T.A., Laird A.R., Ray K.L., Dean Y.M., Glahn D.C., Carter C.S. (2012). Meta-analytic evidence for a superordinate cognitive control network subserving diverse executive functions. Cognitive, Affective, Behav. Neurosci..

[b0390] Noordermeer S.D.S., Luman M., Oosterlaan J. (2016). A systematic review and meta-analysis of neuroimaging in oppositional defiant disorder (ODD) and conduct disorder (CD) taking attention-deficit hyperactivity disorder (ADHD) into account. Neuropsychol. Rev..

[b0395] Odgers C.L., Caspi A., Broadbent J.M., Dickson N., Hancox R.J., Harrington H., Poulton R., Sears M.R., Thomson W.M., Moffitt T.E. (2007). Prediction of differential adult health burden by conduct problem subtypes in males. Arch. Gen. Psychiatry.

[b0400] Odgers C.L., Moffitt T.E., Broadbent J.M., Dickson N., Hancox R.J., Harrington H., Poulton R., Sears M.R., Thomson W.M., Caspi A. (2008). Female and male antisocial trajectories: From childhood origins to adult outcomes. Dev. Psychopathol..

[b0405] Orue I., Andershed H. (2015). The youth psychopathic traits inventory-short version in spanish adolescents—factor structure, reliability, and relation with aggression, bullying, and cyber bullying. J. Psychopathol. Behav. Assess..

[b0410] Orue I., Calvete E., Gamez-Guadix M. (2016). Gender moderates the association between psychopathic traits and aggressive behavior in adolescents. Personality Individ. Differ..

[b0415] Pape L.E., Cohn M.D., Caan M.W.A., van Wingen G., van den Brink W., Veltman D.J., Popma A. (2015). Psychopathic traits in adolescents are associated with higher structural connectivity. Psychiatry Res.: Neuroimag..

[b0420] Perini I., Gustafsson P.A., Hamilton J.P., Kämpe R., Zetterqvist M., Heilig M. (2018). The salience of self, not social pain, is encoded by dorsal anterior cingulate and insula. Sci. Rep..

[b0425] Perneger T.V. (1998). What’s wrong with Bonferroni adjustments. BMJ.

[b0430] Poeppl T.B., Donges M.R., Mokros A., Rupprecht R., Fox P.T., Laird A.R., Bzdok D., Langguth B., Eickhoff S.B. (2019). A view behind the mask of sanity: meta-analysis of aberrant brain activity in psychopaths. Mol. Psychiatry.

[b0435] Prinstein M.J., Dodge K.A. (2008).

[b0440] Puiu A.A., Wudarczyk O., Kohls G., Bzdok D., Herpertz‐Dahlmann B., Konrad K. (2020). Meta-analytic evidence for a joint neural mechanism underlying response inhibition and state anger. Hum. Brain Mapp..

[b0445] Pulkkinen L., Lyyra A.-L., Kokko K. (2009). Life success of males on nonoffender, adolescence-limited, persistent, and adult-onset antisocial pathways: Follow-up from age 8 to 42. Aggressive Behav..

[b0450] Rappaport B.I., Barch D.M. (2020). Brain responses to social feedback in internalizing disorders: a comprehensive review. Neurosci. Biobehav. Rev..

[b0455] Riva P., Romero Lauro L.J., DeWall C.N., Chester D.S., Bushman B.J. (2015). Reducing aggressive responses to social exclusion using transcranial direct current stimulation. Social Cognit. Affective Neurosci..

[b0460] Schriber R.A., Guyer A.E. (2016). Adolescent neurobiological susceptibility to social context. Develop. Cognitive Neurosci..

[b0465] Shaffer D., Fisher P., Lucas C., Dulcan M., Schwab-Stone M. (2000). NIMH diagnostic interview schedule for children version IV (NIMH DISC-IV): description, differences from previous versions, and reliability of some common diagnoses. J. Am. Acad. Child Adolesc. Psychiatry.

[b0470] Simmons A., Matthews S.C., Paulus M.P., Stein M.B. (2008). Intolerance of uncertainty correlates with insula activation during affective ambiguity. Neurosci. Lett..

[b0475] Tabachnick B., Fidell L. (2013).

[b0480] Taber-Thomas B., Pérez-Edgar K. (2015).

[b0485] Tamnes C.K., Herting M.M., Goddings A.-L., Meuwese R., Blakemore S.-J., Dahl R.E., Güroğlu B., Raznahan A., Sowell E.R., Crone E.A., Mills K.L. (2017). Development of the cerebral cortex across adolescence: a multisample study of inter-related longitudinal changes in cortical volume, surface area, and thickness. J. Neurosci..

[bib531] Tielbeek J. (2018). Balancing the Scale of Lady Justice: Biosocial studies of antisocial behavior (Doctoral dissertation).

[b0490] van Baardewijk Y., Stegge H., Bushman B.J., Vermeiren R. (2009). Psychopathic traits, victim distress and aggression in children. J. Child Psychol. Psychiatry.

[b0300] van de Groep I.H., Bos M.G.N., Jansen L.M.C., Achterberg M., Popma A., Crone E.A. (2021). Overlapping and distinct neural correlates of self-evaluations and self-regulation from the perspective of self and others. Neuropsychologia.

[b0205] van Domburgh L. (2009).

[b0495] van Domburgh L., Doreleijers T.A., Geluk C., Vermeiren R. (2011). Correlates of self-reported offending in children with a first police contact from distinct socio-demographic and ethnic groups. Child Adolesc. Psychiatry Mental Health.

[b0500] van Domburgh L., Geluk C., Jansen L., Vermeiren R., Doreleijers T. (2019). Antisocial behavior and victimization over 2-year follow-up in subgroups of childhood arrestees. J. Interpersonal Violence.

[b0505] van Domburgh L., Vermeiren R., Blokland A.A.J., Doreleijers T.A.H. (2009). Delinquent development in dutch childhood arrestees: developmental trajectories, risk factors and co-morbidity with adverse outcomes during adolescence. J. Abnorm. Child Psychol..

[b0510] van Goozen S.H.M., Fairchild G. (2008). How can the study of biological processes help design new interventions for children with severe antisocial behavior?. Dev. Psychopathol..

[b0515] van Heukelum S., Tulva K., Geers F.E., van Dulm S., Ruisch I.H., Mill J., Viana J.F., Beckmann C.F., Buitelaar J.K., Poelmans G., Glennon J.C., Vogt B.A., Havenith M.N., França A.S.C. (2021). A central role for anterior cingulate cortex in the control of pathological aggression. Curr. Biol..

[b0520] Veenstra R., Lindenberg S., Verhulst F.C., Ormel J. (2009). Childhood-limited versus persistent antisocial behavior: why do some recover and others do not? the TRAILS study. J. Early Adolescence.

[b0525] Vetter N.C., Buse J., Backhausen L.L., Rubia K., Smolka M.N., Roessner V. (2018). Anterior insula hyperactivation in ADHD when faced with distracting negative stimuli. Hum. Brain Mapp..

[b0530] Yang Y., Raine A. (2009). Prefrontal structural and functional brain imaging findings in antisocial, violent, and psychopathic individuals: a meta-analysis. Psychiatry Res..

