# Supplementary material for: Resisting aggression in social contexts: The influence of life-course persistent antisocial behavior on behavioral and neural responses to social feedback
Source: Neuroimage Clin. 2022 Feb 26;34:102973. doi: 10.1016/j.nicl.2022.102973 (PMC8892163; doi:10.1016/j.nicl.2022.102973)
Supplement: Supplementary data 1 [file mmc1.docx]

**Supplementary Materials**

Table S1. Attrition analyses

|  | Excluded Sample  (N = 19) | Included Sample  (N = 54) | Statistics |
| --- | --- | --- | --- |
| No. Persisters | 8 | 12 | Χ^2^= 2.28, p = 0.13 |
| Age | M = 26.43, SD = 1.17 | M = 26.29, SD = 1.53 | T(71) = 0.35, p = 0.72 |
| IQ^1^ | M = 93.72, SD = 10.98 | M = 102.89, SD = 13.46 | T(71) = -2.67, p = 0.009 |
| No. Males | 15 | 46 | Χ^2^ < 0.001, p = 1 |
| YPI Callous-Unemotional Traits^2^ | M = 29.82, SD = 4.45 | M = 29.83, SD = 4.42 | T(71) = -0.003, p = 0.99 |
| YPI Grandiose-Manipulative Traits^2^ | M = 27.68, SD = 8.08 | M = 29.72, SD = 9.27 | T(71) = -0.85, p = 0.399 |
| YPI Impulsive-Irresponsible Traits^2^ | M = 32.21, SD = 9.15 | M = 30.20, SD = 7.99 | T(71) = 0.91, p = 0.367 |
| YPI total score^2^ | M = 89.72, SD = 17.51 | M = 89.74, SD = 19.23 | T(71) = -0.005, p = 0.99 |

^1^ Note that for 17 participants (n*_MRIcomplete_* = 3, n*_MRImissing_* = 14), the IQ tests at T5 were not completed, due to time constraints (n*_MRIcomplete_* = 3), or limitations imposed by the COVID-19 pandemic to complete the WAIS in person (n*_MRImissing_* = 14). Therefore, we estimated these scores using multiple imputation (mice package v3.13.0; van Buuren & Groothuis-Oudshoorn, 2011), based on the other variables reported in this table, as well as prior IQ scores (T4).

^2^ Note that for three participants (n*_MRIcomplete_* = 2, n*_MRImissing_* = 1), the YPI was not completed. Therefore, we estimated these scores using multiple imputation, based on the other variables reported in this table, as well as prior IQ scores (T4).

**Table S2**

*Pairwise comparisons using t-tests for confirmatory ROI Analyses: Feedback type (Salience) for ACC, Left and Right Insula, and dlPFC*

|  | ACC | Left Insula | Right Insula | dlPFC |
| --- | --- | --- | --- | --- |
| Pairwise comparison |  |  |  |  |
| Positive vs. Negative | *p* = .043 | *p* = .0393 | *p* = 1.00 | *p* = .020 |
| Positive vs. Neutral | *p* = .017* | *p* = .0019* | *p* = .036 | *p* = .091 |
| Negative vs. Neutral | *p* = 1.00 | *p* = .0902 | *p* = .177 | *p* = 1.00 |

* Statistically significant after bonferroni correction for correlated variables (threshold of α = 0.0287)

**Table S3**

*ANOVA results for the influence psychopathic traits on salience difference scores*

| Predictor | *df_Num_* | *df_Den_* | *F* | *p* |
| --- | --- | --- | --- | --- |
| Callous-Unemotional | 18 | 69 | 1.14 | .331 |
| Grandiose-Manipulative | 29 | 58 | 0.89 | .625 |
| Impulsive-Irresponsible | 32 | 55 | 1.14 | .329 |
| YPI | 47 | 40 | 1.50 | .096 |

*Note.* *df_Num_* indicates degrees of freedom numerator. *df_Den_* indicates degrees of freedom denominator.

**Table S4**

*MINI diagnoses in the persister and desister groups*

|  | Desister (N = 42) | Persister (N = 12) |
| --- | --- | --- |
| MINI Diagnosis |  |  |
| Past Major Depressive Disorder | 6 (14.29%) | 5 (45.45%) |
| Current Mood Disorder due to physical condition | 1 (2.38%) | 0 (0.00%) |
| Past Mood Disorder due to drug use | 0 (0.00%) | 1 (9.09%) |
| Agoraphobia | 1 (2.38%) | 3 (27.27%) |
| Obsessive-Compulsive Disorder | 1 (2.38%) | 1 (9.09%) |
| Generalized Anxiety Disorder | 7 (16.6%) | 3 (27.27%) |
| Alcohol dependence / abuse | 15 (35.71%) | 5 (45.45%) |
| Drug (non-alcohol) dependence / abuse | 10 (23.8%) | 7 (63.6%) |
| Attention Deficit Hyperactivity Disorder | 0 (0.00%) | 1 (9.09%) |
| Posttraumatic Stress Disorder | 1 (2.38%) | 2 (18.18%) |

^1^ Note that screening for behavioral issues and clinical disorders was performed on the same day of the MRI for the controls (using the diagnostic interview), and for participants from the persister/desister group 0-375 days before the MRI session, with an average of 108 days.

**Table S5**

|  | Timepoint | | | | | | | | | | | |
| --- | --- | --- | --- | --- | --- | --- | --- | --- | --- | --- | --- | --- |
|  | T1 | | | | T4 | | | | T5 | | | |
| ***DISC / MINI Diagnosis^1^*** | Desister | Persister | | Total | Desister | Persister | | Total | Desister | Persister | | Total |
| DBD^2^ | 9 | 3 | | 12^3^ | 0 | 4 | | 4 | - | - | | - |
| ASPD | - | - | | - | - | - | | - | 0 | 12 | | 12 |
| ADHD | 4 | 3 | | 7 | 4 | 0 | | 4 | - | - | | - |
| PTSS | - | - | | - | 1 | 0 | | 1 | - | - | | - |
| None | 29 | 6 | | 35 | 29 | 3 | | 32 | - | - | | - |
| Missing | - | - | | - | 8 | 5 | | 13 | - | - | | - |
|  |  |  | |  |  |  | |  |  |  | |  |
| ***Age*** | T0^4^ | | T1 | | T2 | | T3 | | T4 | | T5 | |
| Mean | 10.49 | | 10.9 | | 12.01 | | 13.01 | | 18.11 | | 26.52 | |
| SD | 1.43 | | 1.47 | | 1.53 | | 1.58 | | 1.31 | | 1.63 | |
| Min | 5.95 | | 6.21 | | 7.47 | | 8.37 | | 14.76 | | 21.6 | |
| Max | 11.97 | | 12.78 | | 14.38 | | 15.15 | | 20.38 | | 29.14 | |
|  |  | |  | |  | |  | |  | |  | |
|  | T0 | | | | | | | | | | | |
| ***Type of index crime*** | Desister (n = 42) | | | | Persister (n = 12) | | | | Total (n = 54) | | | |
| Arson | 7 | | | | 0 | | | | 7 | | | |
| Theft | 7 | | | | 1 | | | | 8 | | | |
| Violent crime | 4 | | | | 4 | | | | 8 | | | |
| Public nuisance | 11 | | | | 0 | | | | 11 | | | |
| Vandalism | 8 | | | | 4 | | | | 12 | | | |
| Missing | 5 | | | | 3 | | | | 8 | | | |

^1^ Note that the DISC was only administered at T2 or T3 if it was not administered at T1 ^2^ Means participant had at least a DBD diagnosis ( DBD CD / ODD / CD + ODD / ADHD + OD, ADHD + CD, ADHD + OD + CD)

^3^ Note that of these 12 participants, 7 were diagnosed with DBD before the age of 12, and 5 after the age of 12 (during the current study).

^4^ Note that T0 corresponds to the timepoint at which participants were arrested.


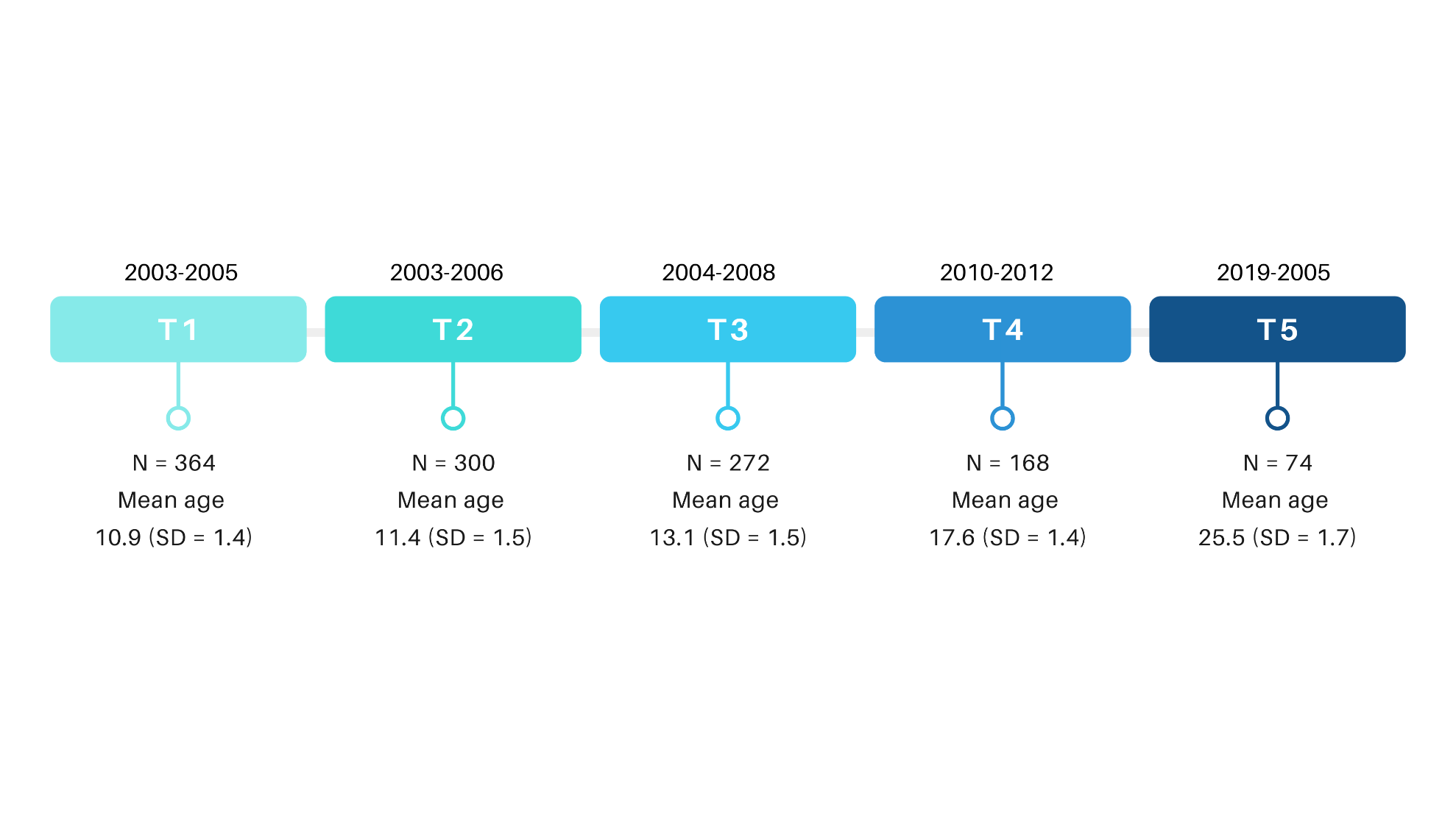


Figure S1. **(A)** Overview of the longitudinal RESIST study (Research on Individual (Anti-) Social Trajectories), aimed at investigating predictors and consequences of (anti-)social behavior across development. **(B)** Participant flow chart diagram for the fifth wave (T5) of the RESIST study.

**Assessed for eligibility (n=255)**

  Participated in T4 (n = 130)

  Did not participate in T4 (n= 125)

**Excluded (n=181)**

  Did not respond (n= 94)

 Declined to participate (n = 13)

  Did not show up (n= 6)

  Deceased (n= 1)

**Home Visit (n=74)**

**Excluded (n=19)**

  MRI contra-indications (n= 1)

  Did not show up (n= 3)

  Declined to participate (n= 15)

**MRI Session (n=55)**

**Original sample (n=364)**

**Persister (n=12)**

**Desister (n=42)**

**Included in Analyses (n=54)**

**Classification**

**Excluded (n=1)**

  Movement > 3mm (n= 1)

**Detailed description ROI Analyses**

To test our second hypothesis about neural activation during the receipt of social feedback, next to the whole-brain analyses described above, we performed three ANOVAs with feedback type (Negative, Positive, Neutral) as independent variable, and ROI parameter estimates for the ACC and left and right Insula as dependent variable. To test for interactions with Group status, this categorical variable was included as a covariate. ANOVAs were followed-up by computing pairwise comparisons between conditions and groups. As an additional control analysis, we explored whether results remained the same when Persist and Desist subgroups were combined in one Group (early antisocial experiences). The ANOVAs with Group (i.e., control, early antisocial experiences) did not differ from the ANOVAs with the pre-registered subgroups (i.e., Control, Persistent, Desistent) for any of the behavioral and neural analyses. Therefore, we only report the analyses with the three groups in the result section.

To test how psychopathic trait scores (Grandiose-Manipulative, Callous-Unemotional, Impulsive-Irresponsible, YPI total) influenced neural activation, we calculated saliency difference scores (i.e., parameter estimates for Positive + Negative > Neutral) for the ACC, left and right Insula and subsequently performed ANOVAs with psychopathic trait scores (Grandiose-Manipulative, Callous-Unemotional, Impulsive-Irresponsible, YPI total) as IVs and saliency difference scores as DVs.


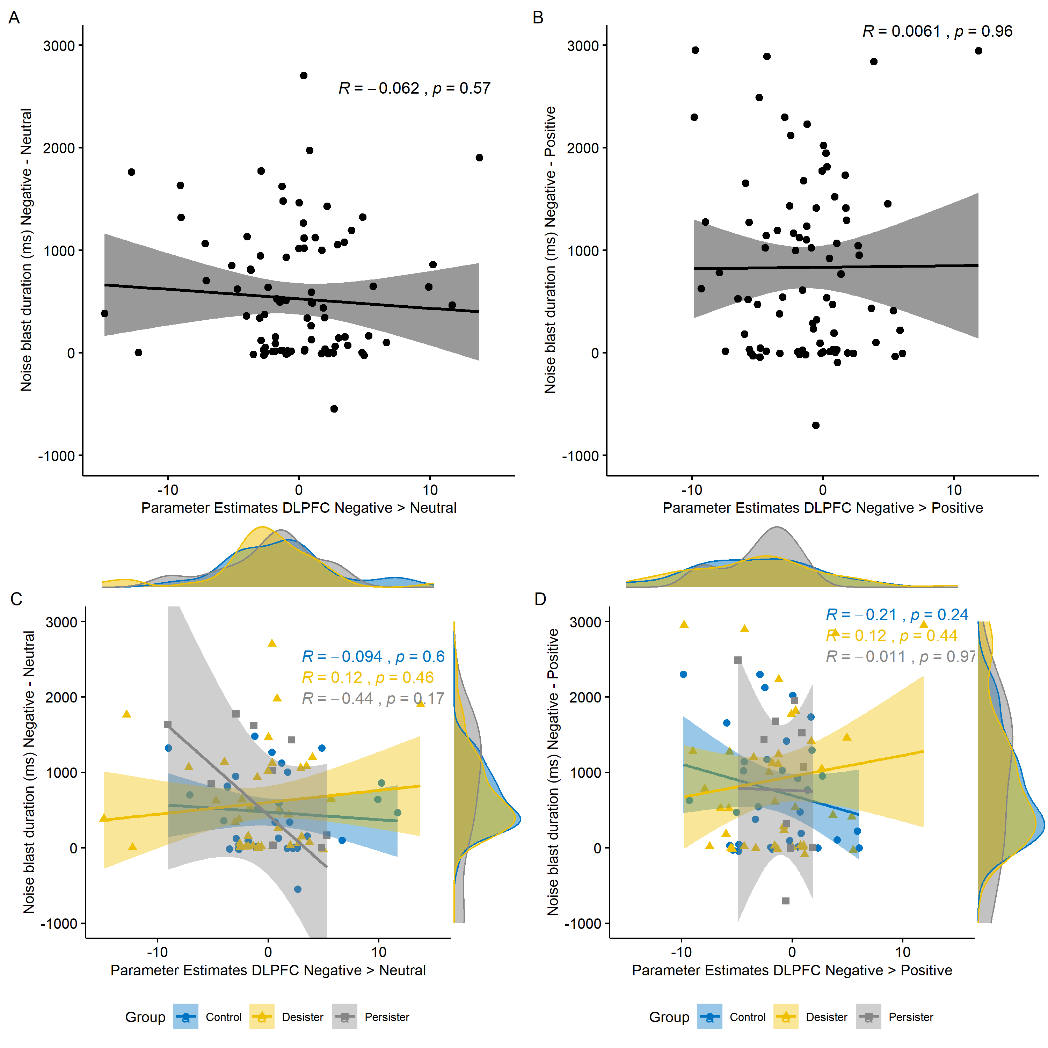


*Figure S2.* **(A)** Brain-behavior association between difference scores in dlPFC activity (Negative > Neutral feedback) and noise blast duration (Negative > Neutral feedback), and **(B)** difference scores in dlPFC activity (Negative > Positive feedback) and noise blast duration (Negative > Positive feedback). Panels **C-D** show the aforementioned associations split per group.
